# Unraveling the Genetic Basis of Cold Tolerance in Peanut (*Arachis hypogaea* L.) via QTL Mapping and Identification of Ahcold18 as a Candidate RWP-RK Transcription Factor

**DOI:** 10.3390/plants15182749

**Published:** 2026-09-08

**Authors:** Yu Zhang, Shunli Cui, Xiukun Li, Mingyu Hou, Yingru Liu, Guoqing Yu, Shutao Yu, Haixin Wang, Puxiang Shi, Hongtao Deng, Lifeng Liu

**Affiliations:** 1 State Key Laboratory of North China Crop Improvement and Regulation, Key Laboratory for Crop Germplasm Resources of Hebei, North China Key Laboratory for Crop Germplasm Resources of Education Ministry, Hebei Agricultural University, Baoding 071001, China; zhangyu611426@163.com (Y.Z.); cuishunli1999@163.com (S.C.); lixiukun1103@163.com (X.L.); houmy@hebau.edu.cn (M.H.); yrliu0917@163.com (Y.L.); hongtaodeng97@163.com (H.D.); 2 Institute of Sandy Land Management and Utilization of Liaoning, Fuxin 123000, China; fuyaomuye@126.com (G.Y.); shutaoyu@163.com (S.Y.); wanghaixin99@163.com (H.W.); spx_104@126.com (P.S.)

**Keywords:** *Arachis hypogaea* L., *Ahcold18*, cold tolerance, QTL mapping, Weighted gene co-expression network, KASP marker

## Abstract

Peanut (*Arachis hypogaea* L.) is a globally vital oil and cash crop. However, frequent cold stress severely compromises its yield stability. To address this challenge, we conducted quantitative trait locus (QTL) mapping for two cold tolerance-associated traits, namely relative emergence rate (RER) and relative emergence index (REI), across four distinct environments using a recombinant inbred line (RIL) population derived from the cold-tolerant landrace Silihong and cold-sensitive cultivar Jinonghei 3. Two core QTLs associated with cold tolerance were detected, including *qRER6* stable across all environments with PVE of 4.91–5.15% and a co-localized QTL *qRER18.1*/*qREI18.2* on chromosome 18 that governs both target traits with PVE of 14.56–14.71% and 10.34%. Through integrated analysis of QTL mapping and Weighted gene co-expression network analysis (WGCNA), *Arahy.657RUG* was identified as a candidate cold tolerance gene and designated *Ahcold18*. Differential expression analysis and heterologous overexpression in *Arabidopsis thaliana* under freezing stress (−9 °C) showed that *Ahcold18* enhances plant survival under low-temperature stress, suggesting a general role in cold tolerance that warrants further investigation in the context of peanut chilling tolerance. A gene-based KASP marker (*KASP-2374669*), developed from variant sites within *Ahcold18*, showed preliminary association with RER and REI in the RIL population; however, further validation in diverse germplasm is required to confirm its utility for marker-assisted selection. This study provides a critical genetic resource and a precise technical tool for marker-assisted breeding of cultivated peanut with cold tolerance.

## 1. Introduction

Peanut (*Arachis hypogaea* L.) is a globally important oilseed and cash crop, contributing substantially to vegetable oil supply and agricultural economic development worldwide [1,2,3]. However, under global climate change, extreme climatic events including low-temperature and drought stresses have become increasingly frequent since the mid-20th century. Recurrent low-temperature stress episodes have been reported worldwide every three to four years, causing 13–15% crop yield losses and posing a severe threat to global food and oil security [4,5]. As a thermophilic species with a minimum germination temperature of 12–15 °C, peanut is highly susceptible to chilling stress. Plants experience two distinct forms of cold stress: chilling (0–15 °C), which disrupts metabolic processes and membrane fluidity, and freezing (<0 °C), which causes ice crystal formation and cellular damage; as a thermophilic species, peanut is primarily challenged by chilling stress during germination and early growth. It has been confirmed that during the seed germination stage under low-temperature conditions, lipid peroxidation induced by the accumulation of free radicals is a critical factor affecting seed vigor. This severely impairs seed germination, seedling establishment, as well as subsequent vegetative and reproductive growth, ultimately leading to yield reduction [6,7]. This thermophilic and cold-sensitive characteristic severely restricts peanut production in cold-prone regions of China, especially in Northeast China, and constitutes the main bottleneck restricting the large-scale development, quality improvement, and efficiency enhancement of the local peanut industry [8,9]. Elucidating the genetic basis and molecular regulatory mechanisms of peanut cold tolerance is not only crucial for understanding the adaptive evolution of peanut to low-temperature environments but also provides essential theoretical support and genetic resources for breeding cold-resistant varieties through molecular marker-assisted selection, which is of great practical significance for safeguarding global peanut production stability [2,3].

The development of cold tolerance in plants constitutes a systemic adaptive process, involving a multi-layered regulatory network that spans from signal perception to physiological adaptation. This process is initiated by cellular perception of temperature reduction, which triggers early events such as calcium signaling [10], and potentially involves integrating environmental cues through sensors like photoreceptors [11]. The cold signal ultimately converges in the nucleus, driving large-scale transcriptional reprogramming. Among the key regulators, the Dehydration-Responsive Element Binding protein 1/C-repeat Binding Factor (DREB1/CBF) family of transcription factors plays a central role by activating the expression of downstream Cold-Regulated (*COR*) genes, thereby enhancing freezing tolerance [12,13]. This pathway is subject to intricate regulation, including transcriptional responses to rapid cooling mediated by calmodulin-binding transcription activators (CAMTAs) [14], perception of gradual temperature decreases by circadian clock components [15], and redox-based modifications [16].

Emerging evidence indicates that plant perception of low temperature and other abiotic stresses centers on a core mechanism residing in the endoplasmic reticulum (ER). Low temperature directly perturbs protein folding homeostasis within the ER, eliciting ER stress. Beyond serving as a ubiquitous cellular stress response, this process functions as a pivotal hub for cold signal sensing and transduction. Subsequently, the ER orchestrates the transduction of stress signals to the nucleus, initiating widespread transcriptional reprogramming [17]. Among transcription factors mediating plant environmental adaptation, the RWP-RK family—characterized by a conserved RWP-RK domain—has recently been shown to regulate heat tolerance in pearl millet (*Pennisetum glaucum*). Specifically, RWP-RK transcription factors co-regulate with heat shock factors (HSFs) and endoplasmic reticulum (ER)-associated genes to facilitate the transcriptional clearance of temperature-induced misfolded proteins during heat stress [18]. Genome-wide analyses indicate that genes of this family are widespread in plants, and their promoter regions are enriched with *cis*-acting elements related to abiotic stress and hormone responses, suggesting they may integrate environmental signals to regulate adaptive responses [19]. However, it remains largely unknown whether and how RWP-RK transcription factors, in particular, directly participate in the low-temperature stress response and couple with protective secondary metabolic pathways—a question that warrants in-depth investigation.

In recent years, research on cold tolerance in major cereal crops such as rice and maize has advanced considerably [20,21]. In contrast, studies on cold tolerance in peanut remain relatively limited. Existing studies have mainly focused on identifying quantitative trait loci (QTLs) and candidate genes associated with cold-responsive pathways [22]. For instance, overexpression of metabolic enzyme genes such as *AhTPPs*, *AhTPS9*, and *AhALDH2B6* has been shown to significantly enhance cold tolerance in peanut [23]. Furthermore, integrated multi-omics analyses, including small RNA sequencing, have identified low-temperature-responsive miRNAs and their regulatory target modules [24]. Currently, research on cold tolerance in peanuts is relatively scarce and mostly limited to single research methods, lacking systematic studies integrating QTL mapping, transcriptome analysis, and gene function verification. In this study, we focus on chilling tolerance during peanut germination (~10 °C), while the heterologous validation in *Arabidopsis* employed freezing stress (−9 °C) to assess the general low-temperature tolerance function of *Ahcold18*. Therefore, this study comprehensively employs QTL mapping in combination with WGCNA to mine candidate genes, perform validation, and develop gene-based KASP markers, in order to provide candidate genes and selective KASP markers for marker-assisted breeding of cold-tolerant peanut varieties and enhance our understanding of the genetic basis of cold tolerance in peanuts.

## 2. Results

### 2.1. Phenotypic Variation in Cold Tolerance-Related Traits in the RILs and Their Parents

**Figure 1:**
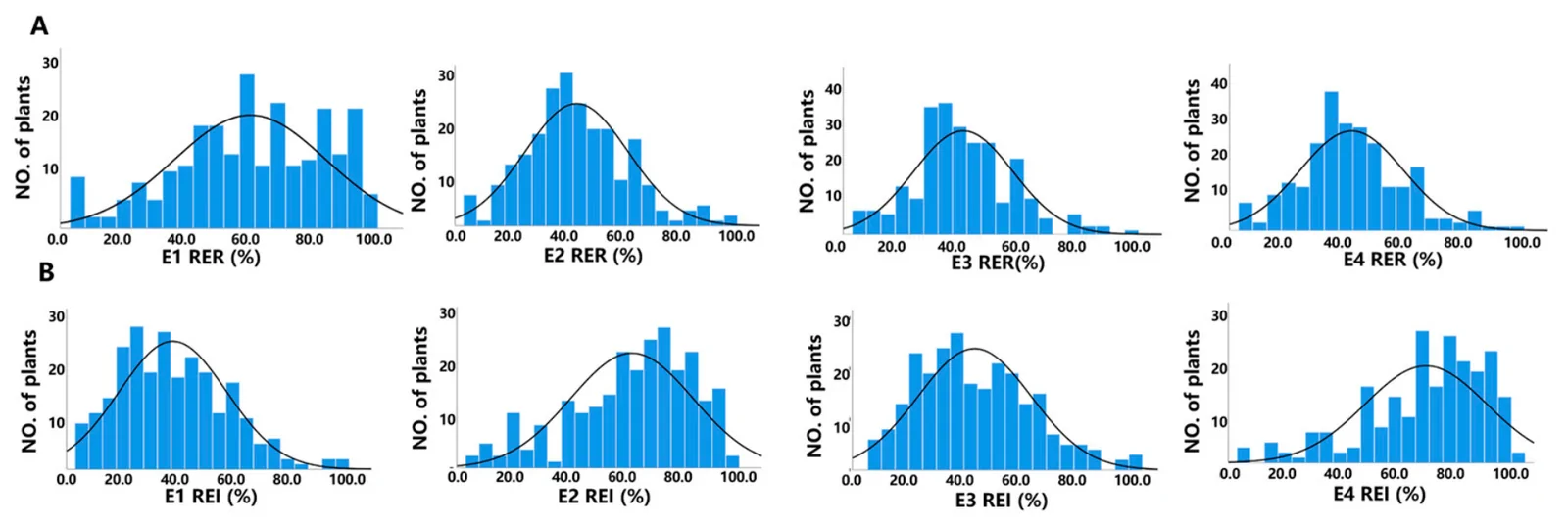
Frequency distribution of cold tolerance-related traits in the RIL population (248 RILs) derived from peanut accessions Silihong and Jinonghei 3 with three replications: (**A**) Combined frequency distribution histograms of relative emergence rate (RER) in the Silihong × Jinonghei 3 RIL population across four field environments: E1 (2021 Baoding, early sowing: 16 April; control sowing: 3 May), E2 (2021 Fuxin, early sowing: 25 April; control sowing: 14 May), E3 (2022 Baoding, early sowing: 15 April; control sowing: 2 May), and E4 (2022 Fuxin, early sowing: 17 April; control sowing: 12 May). Each sub-panel corresponds to one environment, with the *x*-axis showing RER value intervals (midpoints labeled in parentheses). (**B**) Frequency distribution histograms of relative emergence index (REI) in the same RIL population across environments E1–E4 (environmental details as in (**A**)). The *x*-axis shows REI value intervals, with midpoints labeled in parentheses.

A panel of 248 RILs and two parental lines were phenotyped for REI and RER in four environments, respectively. Over the four environments, Silihong exhibited RER of 70.00%, 63.16%, 97.14%, and 72.22%, respectively, in contrast to Jinonghei 3, which showed 45.00%, 18.75%, 43.48%, and 45.46%; for REI, Silihong achieved 60.28%, 70.79%, 83.19%, and 74.35%, while Jinonghei 3 recorded 28.44%, 26.29%, 58.74%, and 37.28% (Table 1; Figure 1). Within the RIL population, RER ranged from 0 to 96% and REI from 0 to 100%, with both traits exhibiting continuous normal distributions (Table 1). Analysis of variance (ANOVA) revealed that genotype (G), environment (E), and their interaction (G × E) all had highly significant effects on both traits (*p* < 0.001; Table 2). Broad-sense heritability estimates were 89.10% for RER and 68.29% for REI, confirming that these traits are predominantly under genetic control, as also supported by the continuous distributions and substantial genetic variance observed. However, the significant G × E interactions indicate that the expression of cold tolerance is modulated by environmental conditions, underscoring the importance of multi-environment testing in breeding programs and suggesting that the genetic architecture of cold tolerance is complex and context-dependent. Collectively, these observations confirm that the RIL population is suitable for QTL mapping, while also warranting a moderated conclusion that the traits are primarily influenced by genetic factors with significant environmental modulation.

### 2.2. QTL Identification for Cold Tolerance-Related Traits

**Figure 2:**
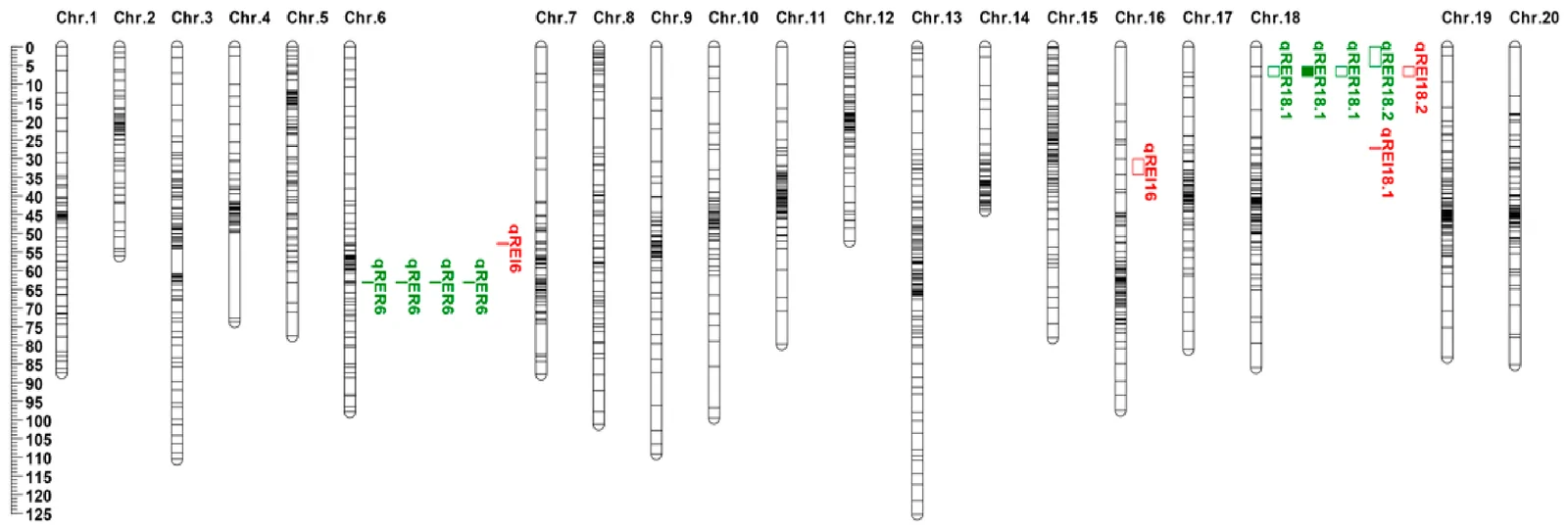
QTL analysis for relative emergence rate (RER) and relative emergence index (REI) under four environments (E1, Baoding 2021; E2, Fuxin 2021; E3, Baoding 2022; E4, Fuxin 2022). Colored boxes represent QTLs detected for the two traits in each environment. The hollow squares represent QTL loci, and their lengths represent the interval sizes of the QTLs

Based on the resequencing-based high-density genetic map and phenotypic data collected from four environments (E1–E4), QTL mapping for RER and REI was conducted using the mixed-model-based composite interval mapping (MCIM) method (Figure 2; Table 3). In total, seven QTLs associated with cold tolerance during peanut germination were detected, distributed on chromosomes 6, 16, and 18, with physical intervals ranging from 552.08 kb to 763.40 kb (average 696.00 kb). The logarithm of odds (LOD) scores ranged from 2.79 to 7.68, the phenotypic variation explained (PVE) from 4.91% to 14.71%, and the additive effects from 1.49 to 7.00.

Among these, three QTLs were associated with RER and mapped to chromosomes 6 and 18. Notably, *qRER6* was consistently detected across all four environments, whereas *qRER18.1* was identified in E1–E3 and *qRER18.2* was specific to E4. The remaining four QTLs were associated with REI and mapped to chromosomes 6, 16, and 18, with PVE of 5.72–10.34%, LOD scores of 3.58–5.97, and additive effects of 1.49–6.64 (Table 3).

Importantly, a co-localized genomic region on chromosome 18, harboring both *qRER18.1* and *qREI18.2*, was identified within marker interval 3611–3612 (Figure 2). This region was consistently associated with RER in E1–E3 and with REI in E3, accounting for 14.56–14.71% and 10.34% of the phenotypic variation, respectively. The overlapping confidence intervals and consistent additive effect directions (with the Silihong allele increasing both traits) suggest that this region may contain a common genetic regulator governing both emergence rate and emergence index under cold stress. We describe this as a “co-localized QTL region” with potential pleiotropic effects, supported by the physical span of 758.53 kb, which is consistent with a single locus exerting effects on both traits.

### 2.3. Weighted Gene Co-Expression Network Analysis (WGCNA) for Low-Temperature Response in Peanut and Identification of Core Cold-Tolerance Modules

**Figure 3:**
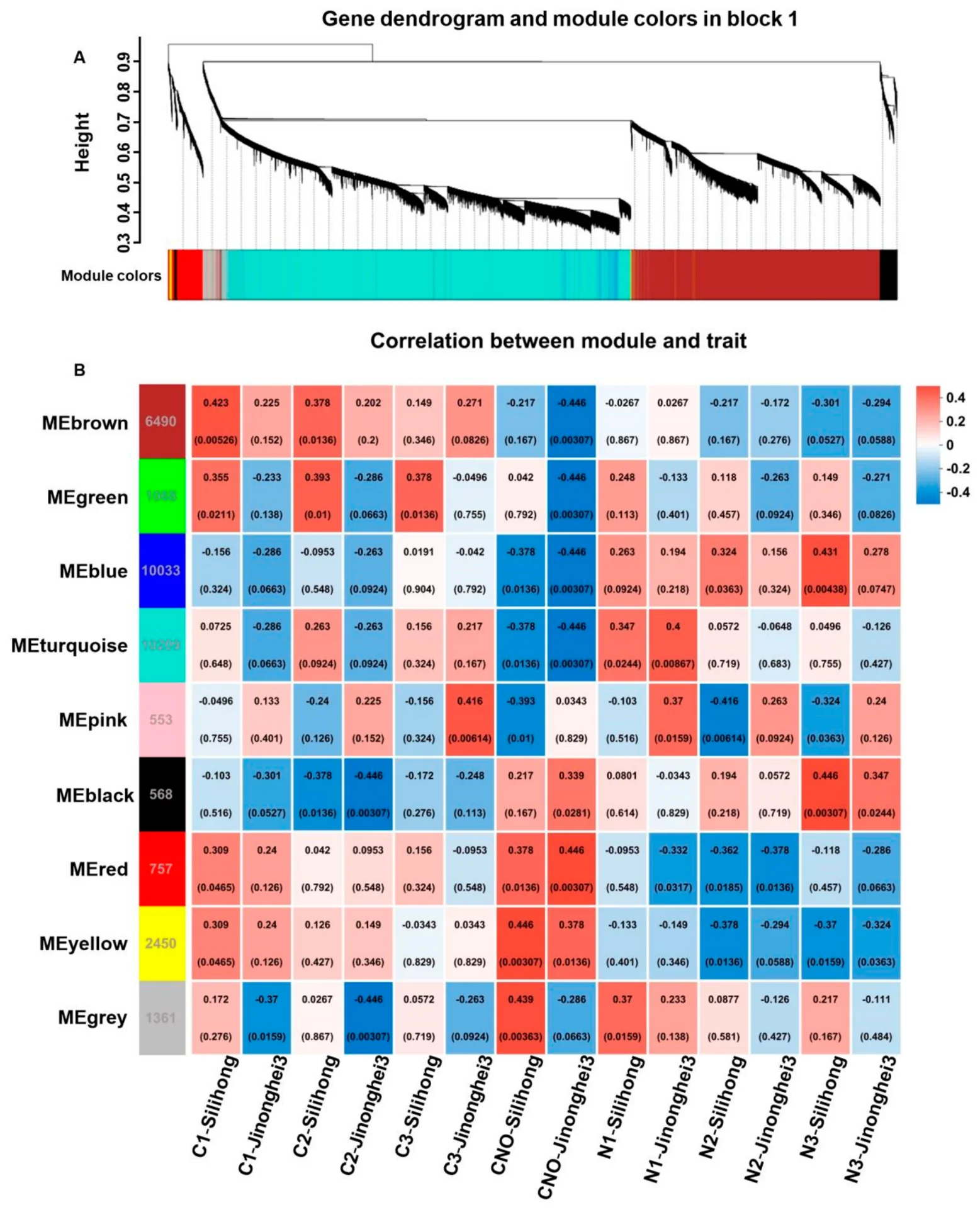
Weighted gene co-expression network analysis (WGCNA) of cold response-related gene modules in *Arachis hypogaea*: (**A**) Hierarchical clustering dendrogram of co-expression modules constructed via WGCNA. The dendrogram reflects the similarity of gene expression patterns; the color bar below the dendrogram represents distinct co-expression modules (each color corresponds to one unique module, e.g., *MEGreen*, *MEBrown*). (**B**) Module–trait correlation heatmap. Each cell displays two values: the upper value is the Pearson correlation coefficient between the module and the trait, and the lower value (in parentheses) is the corresponding *p*-value. The color gradient (from blue to red) indicates the magnitude of the correlation coefficient (blue = negative correlation, red = positive correlation). “C” denotes samples after cold treatment; “N” denotes the normal-temperature control group (15 °C, no cold treatment). Three biological replicates were included for each treatment and time point.

Using 42 transcriptome samples from two parents across four time points and two treatment groups, WGCNA was performed, and 33,489 high-quality expressed genes (FPKM > 1) were selected for network construction. Through soft-thresholding analysis, a power of β = 12 (*R*^2^ = 0.88) was determined, at which the network satisfied the scale-free distribution characteristic. The dynamic tree-cut method clustered all genes into nine co-expression modules with distinct expression patterns (Figure 3). Based on three criteria: (1) significant positive correlation with cold treatment in the cold-tolerant parent Silihong (FDR < 0.05), (2) high module membership (MM > 0.8) indicating strong internal connectivity, and (3) overrepresentation of stress-response Gene Ontology (GO) terms. We identified the MEGreen and MEBrown modules as the core cold-response modules. The MEGreen module exhibited a significant positive correlation with low-temperature treatment in Silihong (r = 0.378, *p* = 0.019), as did the MEBrown module (r = 0.378, *p* = 0.013). Although these correlations were moderate, they were among the highest observed across all modules, and these two were the only modules that consistently showed significant correlation across multiple time points. Furthermore, both modules exhibited at least two-fold higher membership in cold-responsive gene sets compared to other modules, as determined by gene significance (GS) analysis. GO enrichment analysis revealed significant overrepresentation of terms associated with ‘response to stress’ (GO:0006950), ‘cold acclimation’ (GO:0009631), and ‘lipid metabolism’ (GO:0006629) in both modules, which are consistent with known cold-response pathways. Given the previously documented superior cold tolerance of Silihong relative to Jinonghei 3, the moderate correlation values are not unexpected, as cold tolerance is a complex trait likely regulated by multiple independent pathways, and a single module may capture only a portion of the overall response. Collectively, these findings support the role of MEGreen and MEBrown as the core co-expression modules mediating the low-temperature response in peanut.

### 2.4. Identification of Candidate Gene Arahy.657RUG via QTL-Module Integration

**Figure 4:**
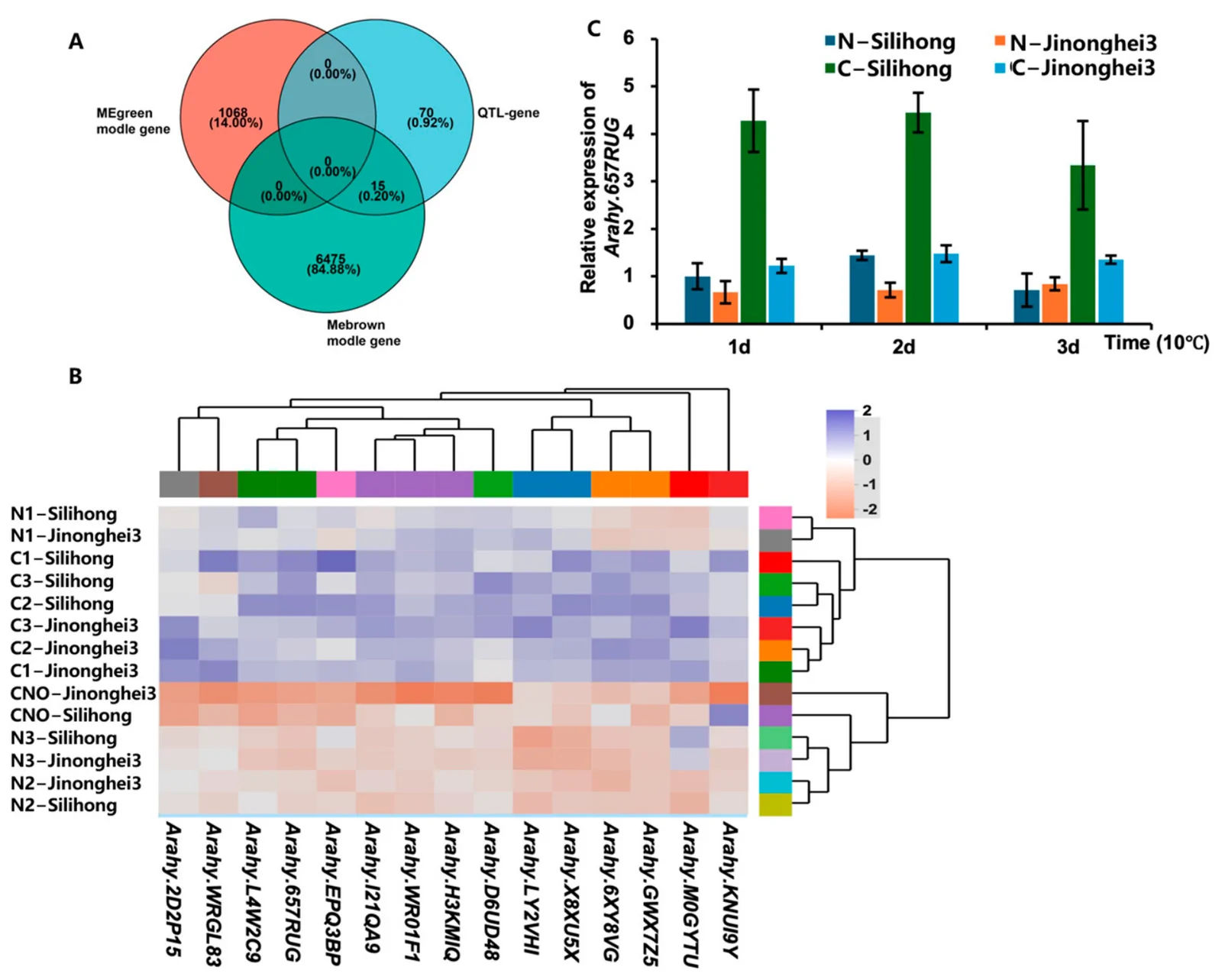
Integrated analysis of co-expression modules, QTL intervals, and *Arahy.657RUG* allele effects on peanut cold tolerance: (**A**) Venn diagram illustrating the gene overlap among the *MEGreen* module, *MEBrown* module, and QTL-associated gene set. (**B**) Heatmap displaying the expression patterns of genes in the co-localized genomic region (of modules and QTL) across samples: N1/N2/N3 (normal temperature, 15 °C) and C1/C2/C3 (low-temperature treatment groups, 10 °C) for accessions Silihong and Jinonghei 3. Color gradients represent normalized gene expression levels. (**C**) Relative expression of *Arahy.657RUG* in Silihong and Jinonghei 3.

Integrated analysis of the MEGreen and MEBrown modules with the previously identified QTL intervals revealed that no genes in the MEGreen module overlapped with *qRER18.1*, whereas 15 genes in the MEBrown module were found to overlap with this cold-tolerance-associated QTL region (Figure 4A). Among these 15 genes, eight contained sequence polymorphisms between Silihong and Jinonghei 3 (Table A3), including SNPs in promoter regions, InDels in 5′ UTRs, nonsynonymous SNPs in exons, and intronic variants.

Cluster analysis of the 15 overlapping genes showed that all were significantly up-regulated following cold stress treatment (Figure 4B; Table A4); of these, 12 genes showed significant differential expression (|log_2_FC| ≥ 1, FDR < 0.05) between the parental lines at one or more time points. However, only *Arahy.657RUG* consistently exhibited significant differential expression across all three time points (1, 2, and 3 days after cold treatment), with fold changes of 3.2×, 4.5×, and 5.1×, respectively (FDR < 0.01 for all time points).

Notably, its module membership (MM) value in the MEBrown module was 0.91, and its gene significance (GS) for cold tolerance was 0.83, further supporting its role as a hub gene within the cold-response network. qRT-PCR validation confirmed that under 10 °C cold treatment, *Arahy.657RUG* expression was significantly higher in Silihong than in Jinonghei 3, corroborating the RNA-seq results.

**Figure A1:**
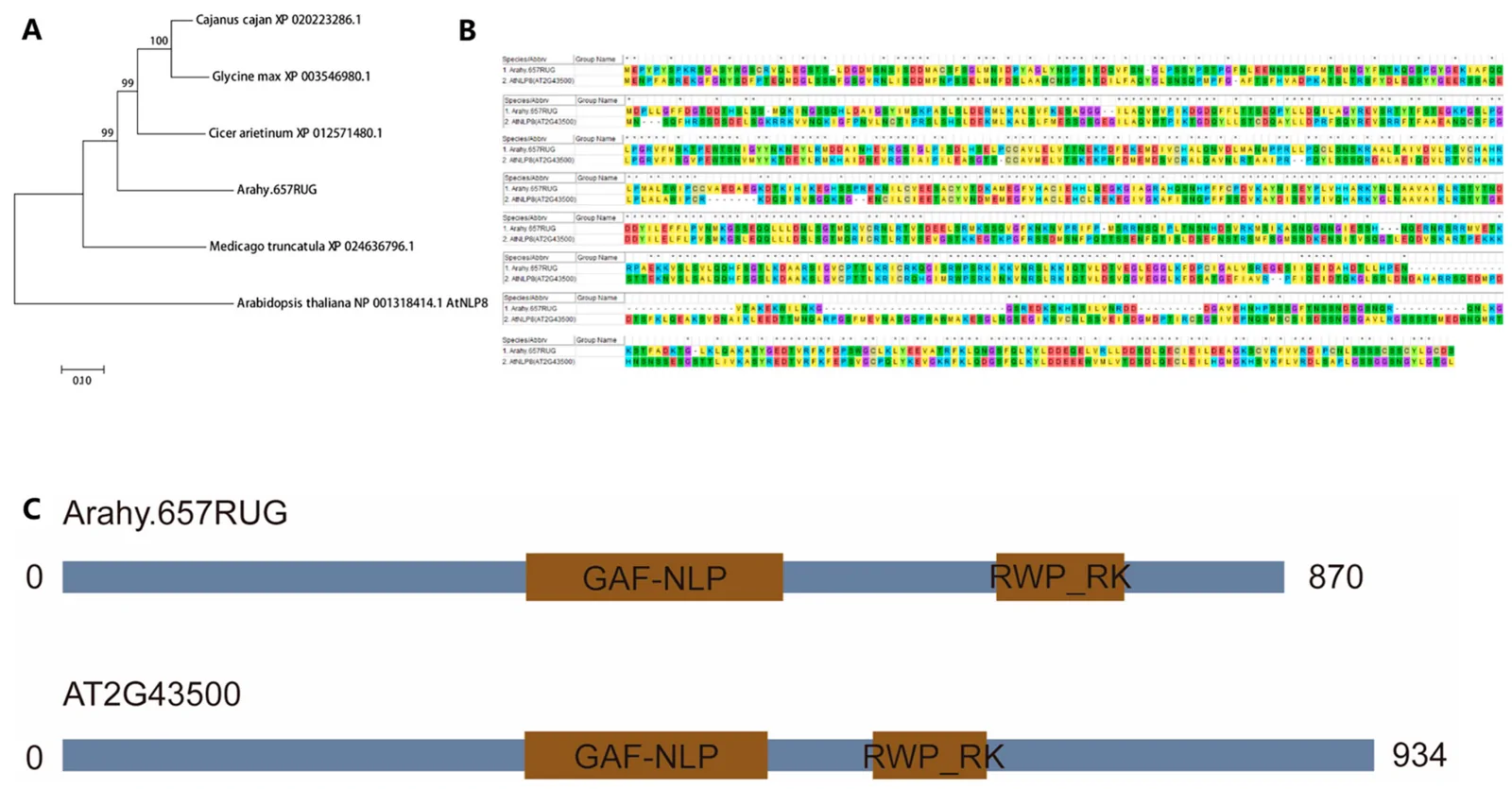
Phylogenetic, sequence homology, and domain analyses of *Arahy.657RUG*: (**A**) Phylogenetic tree based on amino acid sequences showing the evolutionary relationship of *Arahy.657RUG* with homologous proteins from other species. (**B**) Amino acid sequence alignment between *Arahy.657RUG* and *AtNLP8*, highlighting conserved residues. (**C**) Predicted conserved protein domains of *Arahy.657RUG* and *AtNLP8*, revealing functional structural features. The asterisk (*) indicates identical amino acid residues among the compared sequences.

In silico annotation using the TAIR database and BLASTP analysis identified *Arahy.657RUG* as the ortholog of Arabidopsis *NLP8* (*AT2G43500*), with 50.94% amino acid similarity based on sequence alignment (Figure A1B). Phylogenetic analysis revealed homologous genes in several dicot species, including chickpea, soybean, and Arabidopsis (Figure A1A). Domain analysis using InterPro confirmed the presence of both GAF-NLP and RWP-RK domains in *Arahy.657RUG* and *AtNLP8*, which are typical features of NLP family members (Figure A1C). Notably, *AtNLP8* is a transcription factor involved in nitrate signaling and development, but its role in stress responses has not been previously reported. Collectively, these findings implicate *Arahy.657RUG* (hereafter designated as Ahcold18) as a strong candidate gene for cold tolerance during peanut germination, warranting further functional characterization.

### 2.5. Functional Validation of Arahy.657RUG in Arabidopsis

**Figure 5:**
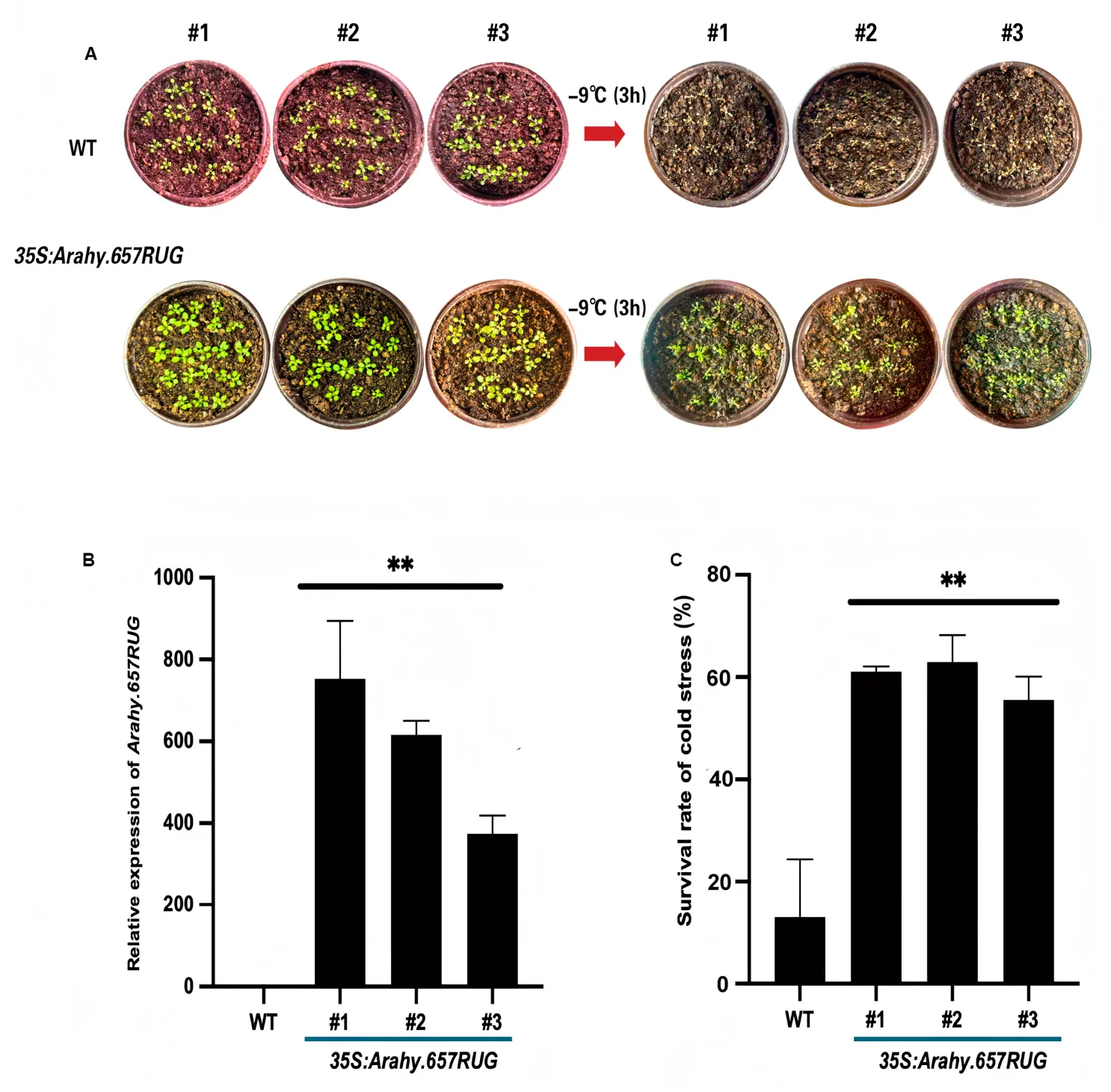
Functional validation of *Arahy.657RUG* alleles in *Arabidopsis thaliana* under low-temperature stress: (**A**) Phenotypic characteristics of Arabidopsis plants following exposure to freezing stress (−9 °C). (**B**) Relative expression level of *Arahy.657RUG* in different lines. Error bars represent SD; ** *p* < 0.01 (one-way ANOVA). (**C**) Survival rate under cold stress in different lines. Error bars represent SD; ** *p* < 0.01 (one-way ANOVA). (**A**–**C**), Wild type (WT), *35S::Arahy.657RUG*; #1, #2, and #3 denote three independent transgenic lines for each construct. Data are presented as mean ± SD from three independent experiments, with 20 plants per line per experiment. ** *p* < 0.01 (one-way ANOVA). The T_3_ homozygous transgenic lines (OE-1, OE-2, OE-3) were used for all cold tolerance assays.

To elucidate the functional role of the candidate gene *Arahy.657RUG* (*Ahcold18*) in peanut cold tolerance, we constructed the overexpression vector 35S::*Ahcold18* using the allele from Silihong and transformed it into *Arabidopsis thaliana*, generating multiple independent transgenic lines. Following exposure to freezing stress at −9 °C for 3 h, the three T_3_ homozygous transgenic lines maintained normal growth, whereas wild-type (WT) plants sustained severe cold damage (Figure 5A). After 10 days of recovery, the mean survival rate of WT across three independent experiments was only 12.50%, while the three transgenic lines exhibited significantly higher survival rates (*p* < 0.01) (Figure 5C). Quantitative real-time PCR confirmed that *Ahcold18* expression was significantly higher in transgenic lines than in WT under cold stress (Figure 5B). While these results suggest that *Ahcold18* contributes to general low-temperature tolerance in a heterologous system, they do not directly validate its role in the chilling tolerance phenotype (approximately 10 °C) originally mapped in peanut. Given the differences in both the stress regime and the species, these findings should be interpreted as supporting evidence for a role in cold stress responses rather than as definitive proof of causality for the chromosome 18 QTL. Additional evidence from fine-mapping, complementation experiments in peanut, or targeted mutagenesis (e.g., CRISPR/Cas9) will be required to establish a causal link between natural variation in *Ahcold18* and the QTL effect.

### 2.6. The Development of Gene-Based KASP Markers for Ahcold18

**Figure 6:**
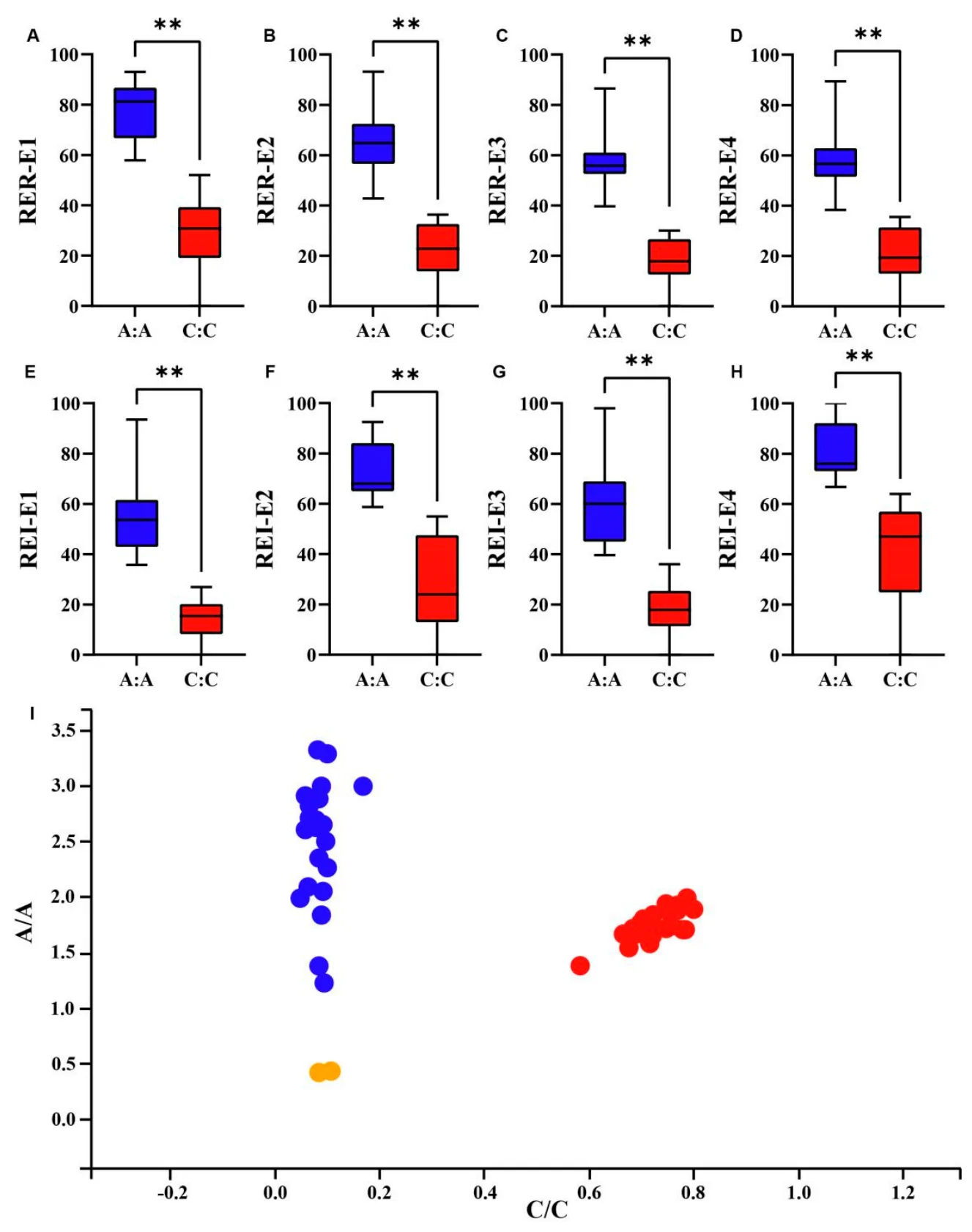
Effect of the KASP-2374669 marker on relative emergence rate (RER) and relative emergence index (REI) in 60 extreme progenies from the RIL population. Lines with the AA genotype showed significantly higher RER and REI than those with the CC genotype (** indicates Student’s *t*-test). The marker was developed from a SNP within Ahcold18 and genotyped in 60 extreme RIL progenies; the association is preliminary and awaits independent validation. (**A**) RER-E1; (**B**) RER-E2; (**C**) RER-E3; (**D**) RER-E4; (**E**) REI-E1; (**F**) REI-E2; (**G**) REI-E3; (**H**) REI-E4; (**I**) genotype cluster.

Within *Ahcold18*, we identified one SNP (Chr.18:2374669, A/C) and two InDel (Chr.18:2374644, C/-; Chr.18:2374903, -/AG), from which gene-based KASP markers were developed. Using 60 extreme progenies (23 cold-tolerant and 37 cold-sensitive) selected from the same RIL population based on the highest and lowest RER values across four environments (E1–E4) (Figure 6), the *KASP-2374669* marker derived from the SNP site successfully genotyped 59 of 60 samples (call rate = 98.3%), distinguishing two homozygous genotypes (AA = 25 lines; CC = 34 lines) with no heterozygous individuals detected, consistent with the RIL status. The AA genotype was present in 19 of 23 cold-tolerant lines (82.6%), while the CC genotype was present in 30 of 37 cold-sensitive lines (81.1%) (Table A5). Across all four environments, lines carrying the AA genotype exhibited significantly higher RER and REI than those with the CC genotype (*p* < 0.01; Benjamini–Hochberg FDR < 0.05). These findings indicate that *KASP-2374669* is significantly associated with cold-tolerance traits during peanut germination and can differentiate materials with contrasting cold sensitivity. However, because this validation was performed using extreme lines derived from the same population used for QTL discovery—which may lead to overestimation due to population structure and linkage disequilibrium—these results should be considered preliminary. Independent validation in diverse genetic backgrounds, including natural populations, breeding lines, and cultivars from different geographic regions, is essential to confirm the marker’s robustness, predictive value, and utility for marker-assisted selection before it can be recommended as a gene-based marker.

## 3. Discussion

QTL mapping serves as a cornerstone approach for dissecting the genetic architecture of complex crop traits and has proven invaluable for improving key agronomic characteristics such as stress tolerance and developmental regulation. This utility is well exemplified by studies on rice cold tolerance, where crossing the cold-tolerant japonica cultivar Taiken 9 (TK9) with the cold-sensitive indica line Taichung Sen 17 (TCS17) led to the identification of the novel major QTL *qCTS11-TK9* [25]. Although QTL mapping has been widely applied to characterize peanut traits including plant architecture, flowering time, oleic acid content, and disease resistance, research focusing on cold tolerance in this crop has remained relatively scarce [26]. To address this gap, we systematically evaluated two key traits, RER and REI, as complementary indicators of cold tolerance at the emergence stage. RER directly reflects the capacity of seeds to overcome cold-induced germination arrest, while REI integrates both the speed and uniformity of emergence under cold stress, providing a more comprehensive measure of germination performance. Both traits exhibited high broad-sense heritability (*H*^2^ = 89.10% for RER and 68.29% for REI), confirming their predominantly genetic control and validating their suitability for QTL mapping. Importantly, we identified a co-localized QTL region, designated *qRER18.1*/*qREI18.2*, that simultaneously governs both traits, suggesting that common genetic mechanisms underlie the regulation of emergence rate and uniformity under chilling conditions. This co-localization implies that selection for one trait may concurrently improve the other, offering a practical advantage for breeding programmers aimed at enhancing overall cold tolerance during peanut germination. Such pleiotropic or tightly linked effects are particularly valuable in crop improvement, as they enable the simultaneous improvement of multiple stress-responsive traits through a single genetic target [27,28]. Thus, the stable QTL identified here not only provides a robust genomic resource for marker-assisted selection but also serves as a starting point for dissecting the molecular networks that integrate the timing, rate, and synchrony of germination under cold stress.

To prioritize candidates among the eight polymorphic genes, we applied three criteria: (1) presence of coding sequence polymorphisms that could affect protein function; (2) consistent differential expression between parental lines across multiple time points under cold stress; and (3) known or predicted functional relevance to stress responses based on gene annotation. Most genes were excluded for the following reasons: four contained only intronic or synonymous variants without predicted functional impact; one showed only transient differential expression; and two had annotations unrelated to stress-response pathways. In contrast, *Arahy.657RUG* uniquely met all three criteria, as it contained both promoter and nonsynonymous coding variants, showed sustained differential expression, and encoded a transcription factor (RWP-RK family) with known regulatory functions.

The RWP-RK family, a plant-specific transcription factor family, has been extensively characterized primarily for nitrogen metabolism regulation (mediated by the NLP subfamily) and reproductive development (governed by the RKD subfamily) [29,30]. Promoters of numerous RWP-RK genes are enriched with abiotic stress-responsive *cis*-acting elements [19]; in *Zanthoxylum armatum*, for instance, family expansion driven by segmental duplication has been implicated in adaptation to drought and other abiotic stresses [31]. More broadly, studies of regulatory gene families in horticultural and model plants—including miRNA-mediated regulatory networks and low-temperature-responsive F-box gene families—have revealed conserved transcriptional and post-translational modules underlying cold acclimation across species [32,33], providing a wider framework in which the cold-responsive role of *Ahcold18* can be interpreted. *Ahcold18* encodes an RWP-RK family transcription factor. Using *Arabidopsis thaliana* as a mediator, we overexpressed *Ahcold18*, and the survival rate of positive seedlings was significantly higher than that of negative seedlings. Based on these published studies and our experimental results, it is considered that *Ahcold18* is associated with cold tolerance in peanut. Next, we need to further investigate the molecular mechanism by which *Ahcold18* enhances cold tolerance in peanut using yeast two-hybrid technology or DAP-seq.

Molecular marker development is one of the most widely used strategies in crop breeding, and numerous markers linked to key agronomic traits have been successfully validated in peanut. Notably, several KASP markers have proven valuable for resistance breeding. For example, markers S4 and S5, tightly associated with the major QTL *qLLS.A02.1*, are effective for late leaf spot resistance [34]. The *AhFAD2C* marker has facilitated efficient selection of high-oleic acid seeds [35]. Additionally, three markers (*SnpAH00614*, *SnpAH00625*, and *SnpAH00626*) have been identified for stem rot resistance via a multi-locus genome-wide association study [36]. By cloning *Ahcold18*, we discovered variant sites within the gene and developed gene-based KASP markers. Preliminary validation showed that this marker is closely associated with RER and REI. Subsequent work will verify the accuracy and broad applicability of this molecular marker for detecting cold tolerance in natural peanut populations, thereby addressing the severe shortage of molecular tools related to cold tolerance in peanuts and providing a powerful resource for accelerating the breeding of cold-tolerant varieties.

However, while fully acknowledging the above findings, several limitations must also be considered. Currently, research on cold tolerance in peanuts remains relatively limited. It has been reported that *AhTPS9*, *AhUGT2*, and *AhUGT268* are all involved in the cold tolerance pathway of peanut [23,27]. From a genetic perspective, cold tolerance in peanut behaves as a quantitative trait governed by multiple loci. Supporting this framework, we identified seven QTLs distributed across chromosomes 6, 16, and 18 that collectively explained 4.91–14.71% of the phenotypic variance. Notably, the co-localized QTL interval harboring *Ahcold18* (*qRER18.1*/*qREI18.2*) was consistently detected across multiple environments (E1–E3). Importantly, multi-environment validation—encompassing two ecologically distinct regions (Baoding and Fuxin) with a 5–6 °C difference in average temperature and approximately 100 mm variation in annual rainfall—confirmed the stability of the identified QTLs. The environmental stability of these QTLs supports their potential use for expanding peanut cultivation into cooler agroecological zones. Supporting this finding, a recent peanut QTL study that utilized RIL populations and a U.S. mini-core collection validated a co-localized QTL region for yield-related traits (HPW, HSW, SP) across diverse environments, with associated KASP markers demonstrating high reliability across distinct genetic backgrounds [28]. However, given the significant genotype-by-environment interactions observed (Table 2), we interpret these findings with caution, as the chromosome 18 region was not detected across all environments—*qRER18.1* was absent in E4, and *qREI18.2* was detected only in E3—indicating that environmental factors may modulate the expression of this locus. Furthermore, we acknowledge that co-localization alone does not definitively distinguish true pleiotropy from the presence of two or more tightly linked genes affecting each trait independently. Therefore, while this co-localized QTL region represents a promising target for candidate gene identification and marker-assisted breeding, fine-mapping is required to resolve the underlying genetic architecture and confirm the causal relationship.

Additionally, more rigorous evaluation is required for the validation of the candidate gene and the marker. While our integrated QTL mapping, WGCNA, expression profiling, and heterologous overexpression data collectively support *Ahcold18* as a strong candidate gene underlying the QTL on chromosome 18, several important caveats must be acknowledged. First, heterologous expression in *Arabidopsis* demonstrates functional potential but does not constitute proof of causality in peanut, and the difference in stress regimes—chilling at ~10 °C in our peanut experiments versus freezing at −9 °C in the *Arabidopsis* assay—means that the mechanisms of chilling and freezing tolerance are partially overlapping but not identical; freezing tolerance additionally involves ice nucleation inhibition, antifreeze proteins, and cryoprotective compounds, so further validation under chilling conditions in peanut or a suitable heterologous system is necessary to confirm the specific role of *Ahcold18* in the phenotype mapped here. Second, the KASP marker KASP-2374669, developed from a variant site within *Ahcold18* (Chr.18:2374669, A/C), showed promising association with RER and REI in our RIL population, but this validation was performed on extreme lines selected from the same population used for QTL discovery, which can lead to overestimation due to population structure and linkage disequilibrium; moreover, the functional effect of the SNP—whether it alters protein structure, expression level, or regulatory activity—remains to be demonstrated, and the marker’s predictive power for breeding applications will depend on its performance across diverse genetic backgrounds, as allele frequencies and linkage relationships may vary among populations. Third, definitive confirmation of *Ahcold18* as the causal gene would require additional approaches, including fine-mapping to narrow the QTL interval, allele-specific functional assays comparing the Silihong and Jinonghei 3 variants, complementation experiments in peanut, or targeted mutagenesis (e.g., CRISPR/Cas9) to demonstrate loss-of-function phenotypes. Therefore, we currently describe *KASP-2374669* as a gene-based QTL-linked KASP marker with potential breeding value and consider *Ahcold18* a strong candidate gene rather than a confirmed causal gene; we emphasize that rigorous validation in independent germplasm panels and further functional studies are essential next steps to fully elucidate its role in cold tolerance and to confirm the marker’s utility for marker-assisted selection.

Thus, the stable QTL identified here not only provides a robust genomic resource for marker-assisted selection but also serves as a starting point for dissecting the molecular networks that integrate the timing, rate, and synchrony of germination under cold stress. Collectively, these findings highlight the chromosome 18 QTL region as a promising target for further functional studies, while also underscoring the necessity of multi-environment validation for practical breeding applications in peanut cold tolerance improvement.

## 4. Materials and Methods

### 4.1. Plant Materials and Field Experiments

A recombinant inbred line (RIL) population derived from a cross between Silihong (female parent, cold-tolerant landrace) and Jinonghei 3 (male parent, cold-sensitive cultivar) was employed in this study. This population was developed via the single-seed descent (SSD) method through successive generations of self-pollination and has been advanced to the F_13_ generation, comprising 248 RILs and two parental lines.

Field experiments were conducted during the 2021 and 2022 growing seasons at two locations: Baoding, Hebei Province (38°40′ N, 115°30′ E) and Fuxin, Liaoning Province (42°01′ N, 121°40′ E), generating four distinct environments: E1 (Baoding, 2021), E2 (Fuxin, 2021), E3 (Baoding, 2022), and E4 (Fuxin, 2022). Two sowing regimes were implemented, with timing strictly determined by soil temperature at 5 cm depth: early sowing was initiated when soil temperature reached approximately 10 °C for three consecutive days, whereas normal sowing occurred when the temperature stabilized at about 15 °C for the same duration. Accordingly, the specific sowing dates were: Baoding 2021—early sowing on 16 April and normal on 3 May; Fuxin 2021—25 April and 14 May; Baoding 2022—15 April and 2 May; Fuxin 2022—17 April and 12 May. Meteorological data for both sites during the germination period (April–May) were obtained from local agrometeorological stations. In Baoding (2021), the mean temperature during the April–May period was 18.4 °C (range: 7.9–32.8 °C), with cumulative precipitation of 6.0 mm and a mean relative humidity of 40.0%. In Fuxin (2021), the mean temperature was 16.4 °C (range: 0.4–30.9 °C), with cumulative precipitation of 26.0 mm and a mean relative humidity of 45.5%. The corresponding values for Baoding 2022 were 18.6 °C (range: 6.4–33.7 °C), 36.5 mm, and 47.0% relative humidity; and for Fuxin 2022 were 16.4 °C (range: 1.3–32.4 °C), 18.6 mm, and 38.2% relative humidity. Detailed daily meteorological records are provided in Table A6.

### 4.2. Phenotype and Statistical Analysis of RER and REI for RIL Population

A randomized complete block design (RCBD) with three replications was adopted across four environments (E1–E4), comprising two parental lines and 248 RILs. Phenotypic data were collected for emergence rate (ER) and emergence index (EI) calculated as ER = (emerged seedlings/total seeds sown) × 100% and EI = ∑(S_i_/D_i_), where S_i_ is the number of seedlings on the i-th day and D_i_ is days after sowing [37,38]. RER and REI under cold stress were calculated as the ratio of the value under low-temperature treatment to that under normal-temperature control, expressed as a percentage [37]. Descriptive statistics (mean, standard deviation, coefficient of variation, skewness, and kurtosis) were computed using SPSS Statistics version 26.0 (IBM Corp.). ANOVA was performed using the general linear model (GLM) procedure in SAS version 9.4 (SAS Institute Inc.), with environment, genotype, and their interaction treated as fixed effects. Broad-sense heritability (H^2^) on a line-mean basis was estimated as *H*_b_^2^ = σ^2^_G_/(σ^2^_G_ + σ^2^_GE_/e + σ^2^_e_/(re)), where σ^2^_G_ is genetic variance, σ^2^_GE_ is genotype × environment interaction variance, σ^2^_e_ is error variance, e = 4 environments, and r = 3 replications. Variance components were estimated using the restricted maximum likelihood (REML) method implemented in R version 4.1.0 with the ‘lme4’ package. This line-mean-based heritability accounts for multiple environments and replications, providing a measure of the reliability of phenotypic assessments for selection purposes.

### 4.3. QTL Mapping

Based on the high-density genetic map previously constructed by the Peanut Research Team of Hebei Agricultural University using resequencing technology [37], which comprised 4499 SNP markers distributed across 20 linkage groups with an average density of 0.38 cM, QTL mapping was performed with QTL IciMapping version 4.0 (https://isbreeding.caas.cn/rj/QTLIciMapping/, accessed on 3 May 2025) using the mixed-model-based composite interval mapping (MCIM) method under parameters of walking speed = 1.0 cM, PIN = 0.001, window size = 10 cM, and forward-and-backward regression for cofactor selection; markers with >10% missing data and those showing significant segregation distortion (*p* < 0.001) were excluded. To determine significance thresholds, 1000 permutation tests were conducted for each trait in each environment (E1–E4), giving environment-specific LOD cutoffs (RER: 2.47, 2.53, 2.51, 2.49; REI: 2.52, 2.48, 2.54, 2.50 across E1–E4), and a unified LOD threshold of 2.5 was adopted as the approximate 95th percentile for consistent QTL identification. QTL nomenclature followed the rule: “q” + trait abbreviation (RER or REI) + chromosome number, with an appended Arabic numeral if multiple QTLs for the same trait were detected on the same chromosome. Mapping was carried out separately for each environment, and QTLs detected in at least two environments or in joint analysis were considered stable; however, the significant genotype-by-environment interaction observed (Table 2) was taken into account when interpreting stability, and our conclusions about QTL stability have accordingly been moderated.

### 4.4. RNA-Seq Analysis and the Weighted Gene Co-Expression Network Analysis (WGCNA)

For transcriptome analysis, the parental lines of the recombinant inbred line (RIL) population—‘Silihong’ (cold-tolerant) and ‘Jinonghei 3’ (cold-sensitive)—were selected as experimental materials. Based on an extensive literature review, systematic meteorological data analysis, and field measurements of soil temperature at 5 cm depth after sowing in 2021 and 2022, an indoor low-temperature identification method was established with two treatments: a low-temperature treatment (daily temperature cycle at an average of 10 °C for 3 consecutive days) and a normal-temperature control (15 °C for the same 3-day period). Relative humidity was maintained at 75% for both treatments, with equal volumes of water supplied at regular intervals. Each treatment included three biological replicates per family, with 20 seeds per replicate.

Peanut embryos were sampled at 0 h (untreated baseline, collected before treatment initiation), 24 h, 48 h, and 72 h post-treatment initiation under both conditions. The 0 h samples were common to both treatments and served as the baseline reference. Samples were coded as follows: “C” for cold treatment, “N” for normal temperature; numbers “1, 2, 3” correspond to 24 h, 48 h, and 72 h, respectively; untreated 0 h samples were designated “DZ0”. For each genotype, treatment, and time point (24, 48, 72 h), three biological replicates were collected, yielding 2 genotypes × 2 treatments × 3 time points × 3 replicates = 36 samples; plus 0 h baseline samples from each genotype in triplicate (2 × 3 = 6), giving a total of 42 samples.

Total RNA was extracted from seed embryos using the Eastep^®^ Super Total RNA Extraction Kit (Promega, Madison, WI, USA). RNA purity and concentration were assessed using a NanoPhotometer^®^ spectrophotometer (IMPLEN, Munich, Germany) and a Qubit^®^ RNA Assay Kit with a Qubit 2.0 Fluorometer (Life Technologies, Carlsbad, CA, USA); RNA integrity was evaluated with an Agilent 2100 Bioanalyzer, and samples with RIN ≥ 7.0 were used for library construction. cDNA libraries were prepared using the NEBNext^®^ Ultra™ RNA Library Prep Kit for Illumina^®^ (New England Biolabs (NEB)—Ipswich, MA, USA) and sequenced on the Illumina NovaSeq 6000 platform with paired-end 150 bp reads. Library preparation and sequencing were performed by Shanghai Majorbio Bio-pharm Technology Co., Ltd. (Shanghai, China).

Raw reads were quality-filtered using fastp version 0.20.0 (removal of adapters, reads with >10% ambiguous bases, and reads with >50% low-quality bases with Q < 20). Clean reads were aligned to the peanut reference genome *Arachis hypogaea cv. Tifrunner* version 2.0 (https://www.peanutbase.org/, accessed on 3 May 2025) using HISAT2 version 2.1.0 with default parameters. Gene expression levels were quantified with StringTie version 2.1.4 and calculated as fragments per kilobase of transcript per million mapped reads (FPKM). For subsequent analyses, genes with mean FPKM < 1 across all samples were filtered out, retaining 33,489 genes; the FPKM values were then normalized using the variance-stabilizing transformation (VST) implemented in DESeq2 version 1.30.0. Differential expression analysis was performed with DESeq2 using thresholds of |log_2_ (fold change)| ≥ 1 and adjusted *p*-value < 0.05.

WGCNA was performed using the WGCNA package version 1.70 in R software version 4.1.0 (R Core Team, 2021, Vienna, Austria). Genes with mean FPKM < 1 across all samples were filtered out, retaining 33,489 high-quality expressed genes for network construction, and the expression matrix was normalized using a variance-stabilizing transformation (VST). A signed network was constructed based on Pearson correlation coefficients to measure gene co-expression similarity. The soft-thresholding power (β) was selected by fitting the network to a scale-free topology model; β = 12 was chosen (R^2^ = 0.88). The topological overlap matrix (TOM) was derived from the adjacency matrix, and hierarchical clustering was performed using average linkage. Modules were identified using the dynamic tree-cutting algorithm with parameters: minimum module size = 30 genes, deepSplit = 2, and mergeCutHeight = 0.25 for merging similar modules. Outlier samples were assessed using the sampleDendrogram function; no samples exceeded 2 standard deviations from the mean connectivity and were thus retained. Module eigengenes (first principal component of each module) were calculated, and module–trait correlations were evaluated via Pearson correlation. To correct for multiple testing, the Benjamini–Hochberg false discovery rate (FDR) procedure was applied, and modules with FDR < 0.05 were considered significantly associated with cold treatment.

### 4.5. Quantitative Real-Time PCR

Total RNA (1 μg per sample) was reverse-transcribed into first-strand cDNA using the PrimeScript™ RT Reagent Kit with gDNA Eraser (TAKARA, Kusatsu, Japan). Quantitative real-time PCR (qRT-PCR) was performed using SYBR Green Master Mix (Vazyme, Nanjing, China) on a LightCycler^®^ 96 instrument (Roche, Basel, Switzerland). Each sample was analyzed in triplicate, with three biological and three technical replicates. The thermal cycling protocol consisted of an initial denaturation step at 95 °C for 10 min, followed by 40 cycles of denaturation at 95 °C for 10 s, annealing at 57 °C for 10 s, and extension at 72 °C for 10 s. Following amplification, a melting curve analysis was conducted by heating to 95 °C for 10 s, cooling to 60 °C for 60 s, and then heating to 97 °C for 1 s. Relative gene expression levels were calculated using the 2^−ΔΔCt^ method and normalized to the internal control gene, Actin. Gene-specific primers used for qRT-PCR are provided in Table A1.

### 4.6. Cold-Tolerance Assay of Transgenic Arabidopsis

Total RNA was isolated from the prepared samples and subsequently reverse-transcribed into first-strand cDNA using the PrimeScript™ RT Reagent Kit with gDNA Eraser (TAKARA, Kusatsu, Japan). The target gene fragment was amplified via high-fidelity polymerase chain reaction (PCR) with PrimeSTAR^®^ HS DNA Polymerase (TAKARA, Kusatsu, Japan), using gene-specific primers whose sequences are detailed in Appendix A. The purified PCR product was then inserted into the pMDC43 overexpression vector through a recombination reaction mediated by the In-Fusion^®^ HD Cloning Kit (TAKARA, Kusatsu, Japan). The recombinant plasmid construct was introduced into *Arabidopsis thaliana* via the floral-dip transformation method, and transgenic plants harboring the target fragment were further screened and identified on a selective medium supplemented with hygromycin.

Hygromycin-resistant positive transgenic seedlings were carefully transferred to plastic pots containing a substrate mixture of potting soil and vermiculite at a volume ratio of 1:2, followed by a 15-day cultivation period under standard growth conditions. For the cold stress treatment, the well-established seedlings were exposed to a low-temperature environment of −9 °C for 3 h, and then transferred back to normal growth temperatures for a 10-day recovery period. After the recovery phase, the survival status of the treated plants was investigated, and the corresponding survival rates were calculated and recorded for subsequent statistical analysis.

### 4.7. KASP Marker Development

Fresh leaves were collected from the RIL population. Based on phenotypic data, extreme offspring within the population were selected. Genomic DNA was extracted from the fresh leaves using the CTAB method, and equal amounts of DNA were obtained from each plant. The purity and concentration of the extracted DNA were assessed, ensuring an OD_260/280_ ratio between 1.8–2.0. The DNA was then diluted to 50 ng/µL as a working solution and stored at −20 °C. Marker development was performed based on the mutation sites within *Ahcold18*. Primers for KASP (Kompetitive Allele-Specific PCR) markers were designed using Primer5 software [39], targeting the 200 bp upstream and downstream regions of the selected sites. The primer sequences are provided in Table A1, and the primers were synthesized by Shanghai Sangon Biotech Co., Ltd. (Shanghai, China). Genotyping was carried out using the KASP detection platform from LGC Genomics. The resulting KASP data were used to analyze the correlation between SNPs and the cold-resistance phenotype in the RILs. It should be noted that the *KASP-2374669* marker was evaluated solely within the RIL mapping population used for QTL discovery. Independent validation in a broader germplasm panel, including diverse genetic backgrounds and breeding lines, is planned and will be essential before this marker can be recommended for routine marker-assisted selection.

## 5. Conclusions

In this study, a strong candidate gene (*Ahcold18*) for cold tolerance during peanut germination was identified through integrated QTL mapping and WGCNA. Heterologous overexpression in *Arabidopsis thaliana* indicated that the peanut *Ahcold18* allele (encoding an RWP-RK transcription factor) from Silihong can improve plant cold survival ability under cold stress. These results extend the known biological functions of the RWP-RK family proteins beyond growth, development, and nutrient homeostasis, suggesting a potential regulatory role in plant cold acclimation. From Ahcold18 variants, we developed the gene-based KASP marker *KASP-2374669*, which was preliminarily associated with RER and REI in the RIL population, but its usefulness for marker-assisted selection awaits validation across diverse germplasm. These findings enrich the current understanding of the molecular basis of cold adaptation during the peanut germination stage under variable climatic conditions, and provide gene and molecular marker resources for future cold-tolerant peanut breeding in response to the increasing frequency of cold injury driven by climate change.

## Figures and Tables

**Table 1:** Phenotypic Variation In Cold Tolerance-Related Traits (RER and REI) in Peanut Parental Lines and RIL Population Across Multiple Environments.

Trait	Environment	Silihong (%)	Jinonghei 3 (%)	RIL Population					
				Range (%)	Mean (%)	SD (%)	CV (%)	Skewness	Kurtosis
RER	E1	70.00	45.00	0–94.00	55.98	24.60	43.94	−0.38	−0.58
RER	E2	63.16	18.75	0–95.00	38.77	18.79	48.47	0.43	0.30
RER	E3	97.14	43.48	0–94.00	36.26	17.33	47.79	0.44	0.48
RER	E4	72.22	45.46	0–96.00	37.10	18.04	48.63	0.43	0.45
REI	E1	60.28	28.44	0–94.00	33.15	19.61	59.16	0.55	0.09
REI	E2	70.79	26.29	0–93.00	58.20	22.28	38.28	−0.69	−0.24
REI	E3	83.19	58.74	0–98.00	38.91	20.60	52.94	0.50	−0.21
REI	E4	74.35	37.28	0–100.00	66.32	22.13	33.37	−0.93	0.47

E1 (Baoding, 2021), E2 (Fuxin, 2021), E3 (Baoding, 2022), and E4 (Fuxin, 2022).

**Table 2:** Analysis of variance for RER and REI in the RIL population grown in multiple environments.

Trait	Source	Df	SS	MS	F-Value	*H*^2^ (%)
RER	Environment (E)	3	308,239.58	102,746.53	2821.60 ***	89.10
	Genotype (G)	247	2,350,391.95	9515.76	261.31 ***	
	G × E interaction	741	768,732.43	1037.43	28.49 ***	
	Error	992	36,123.00	36.41		
REI	Environment (E)	3	20,001.21	6667.07	226.85 ***	68.29
	Genotype (G)	247	2,216,564.17	8973.94	305.34 ***	
	G × E interaction	741	2,108,591.04	2845.60	96.82 ***	
	Error	992	29,155.00	29.39		

*** means significant at *p* < 0.001.

**Table 3:** Co-localized QTL region or SNP for RER and REI across multiple environments.

QTL	Chr.	Position (cM)	Marker Interval	Physical Position (bp)	Physical Interval (Kb)	Trait (Environment)	LOD	PVE (%)	Add	Left CI	Right CI
*qRER6*	6	63	1046–1045	100,708,169–101,385,709	677.54	RER (E1, E2, E3, E4)	2.79–2.92	4.91–5.15	3.71–4.14	62.50	63.50
*qRER18.1*	18	6	3611–3612	2,262,150–3,020,675	758.53	RER (E1, E2, E3)	7.57–7.68	14.56–14.71	6.36–7.00	5.50	7.50
*qRER18.2*	18	3	3610–3611	1,498,753–2,262,150	763.40	RER (E4)	7.52	14.55	6.94	0.00	4.50
*qREI6*	6	53	900–901	13,519,064–14,266,976	747.91	REI (E3)	3.58	5.72	1.49	52.50	53.50
*qREI16*	16	34	3055–3056	7,064,423–7,616,501	552.08	REI (E4)	4.09	7.46	6.05	31.50	36.50
*qREI18.1*	18	27	3616–3617	5,800,863–6,415,056	614.19	REI (E1)	5.79	10.25	6.64	25.50	27.50
*qREI18.2*	18	6	3611–3612	2,262,150–3,020,675	758.53	REI (E3)	5.97	10.34	2.00	5.50	7.50

E1 (Baoding, 2021), E2 (Fuxin, 2021), E3 (Baoding, 2022), and E4 (Fuxin, 2022).

## Data Availability

All of the data generated or analyzed during this study are included in this published article. All data generated or analyzed during this study are included in this published article and its Appendix A. The sequence polymorphisms between parental lines for the 15 candidate genes overlapping the QTL region are provided in Table A3 (Appendix A). The detailed daily meteorological records for both locations (Baoding and Fuxin) across the two growing seasons (2021 and 2022) are compiled in Table A6 (Appendix A). The expression matrix and differential expression results are provided in Table A4. The genotype data for all SNP markers and the KASP genotyping results are provided in Table A5. The high-density genetic map and the complete phenotypic data (including replication-level data for individual RILs across all environments) are available upon request from the corresponding author. All data will be made publicly available upon publication.

## Abbreviations

KASP	Kompetitive allele-specific PCR
RER	Relative emergence rate
REI	Relative emergence index
RIL	Recombinant inbred line
QTL	Quantitative trait loci
SNP	Single nucleotide polymorphism
WGCNA	Weighted gene co-expression network analysis

## Appendix A

**Table A1:** Primer information for real-time quantitative analysis, vector construction, and KASP marker development.

Primer Name	Sequence (5′-3′)
*qRT-657RUG-F*	CTGGTTCAAACCAGAGGCAGAAC
*qRT-657RUG-R*	GAAACGACCCATTTTGTAATTTGAAC
*AhActin-qRT-F*	TTGGAATGGGTCAGAAGGATGC
*AhActin-qRT-R*	AGTGGTGCCTCAGTAAGAAGC
*AtActin-qRT-F*	TCCATGAAACAACTTACAACTCCA
*AtActin-qRT-R*	CGTACTCACTCTCGCAAATCCACA
*Over-657RUG-F*	TGAACTATACAAAGGCGCGCCAATGGAACCATACCCTTATTCTCCC
*Over-657RUG-R*	CGCTCTAGAACTAGTTAATTAACTATGAGTCACATCCTAGGTAGC
*KASP-geneG-X*	GAAGGTGACCAAGTTCATGCTAAATGCATTATCATGCTTCTC
*KASP-geneG-Y*	GAAGGTCGGAGTCAACGGATTAAATGCATTATCATGCTTCTA
*KASP-geneG-C*	TCATGCTCCTATGTTTCATTTGCAT

**Table A2:** Integrated analysis of gene lists from QTL and WGCNA.

Gene ID	Gene Name	Gene Description
*arahy.Tifrunner.gnm2.ann1.2D2P15*	*2D2P15*	MSF1-like family protein
*arahy.Tifrunner.gnm2.ann1.657RUG*	*657RUG*	Plant regulator RWP-RK family protein
*arahy.Tifrunner.gnm2.ann1.6XY8VG*	*6XY8VG*	protein FAR1-RELATED SEQUENCE 12-like isoform X2 [Glycine max]
*arahy.Tifrunner.gnm2.ann1.D6UD48*	*D6UD48*	electron-transfer flavoprotein:ubiquinone oxidoreductase
*arahy.Tifrunner.gnm2.ann1.EPQ3BP*	*EPQ3BP*	uncharacterized protein LOC100806339 [Glycine max]
*arahy.Tifrunner.gnm2.ann1.GWX7Z5*	*GWX7Z5*	hydroxyacylglutathione hydrolase
*arahy.Tifrunner.gnm2.ann1.H3KMIQ*	*H3KMIQ*	Ethylene insensitive 3 family protein
*arahy.Tifrunner.gnm2.ann1.I21QA9*	*I21QA9*	xanthine dehydrogenase 1
*arahy.Tifrunner.gnm2.ann1.KNUI9Y*	*KNUI9Y*	ATPase family AAA domain-containing protein 1-like [Glycine max]
*arahy.Tifrunner.gnm2.ann1.L4W2C9*	*L4W2C9*	AP2-like ethylene-responsive transcription factor ANT-like [Glycine max]
*arahy.Tifrunner.gnm2.ann1.LY2VHI*	*LY2VHI*	Pre-mRNA-splicing factor ATP-dependent RNA helicase
*arahy.Tifrunner.gnm2.ann1.M0GYTU*	*M0GYTU*	mitochondrial substrate carrier family protein C-like [Glycine max]
*arahy.Tifrunner.gnm2.ann1.WR01F1*	*WR01F1*	C-terminal domain phosphatase-like 4
*arahy.Tifrunner.gnm2.ann1.WRGL83*	*WRGL83*	UDP-Glycosyltransferase superfamily protein
*arahy.Tifrunner.gnm2.ann1.X8XU5X*	*X8XU5X*	uncharacterized protein LOC100801419 isoform X1 [Glycine max]

**Table A3:** Sequence polymorphisms between parental lines Silihong and Jinonghei 3 within candidate genes overlapping the QTL region on chromosome 18.

Gene	Gene Position (bp)	SNP Position (bp)	Jinonghei 3	Jinonghei 3	Location	Indel Position (bp)		Jinonghei 3	Jinonghei 3	Location
657RUG	2,371,561–2,377,933	2,374,669	A	C	Intron	2,374,644	2,374,644	C	-	Intron
						2,374,903	2,374,903	-	AG	Intron
H3KMIQ	2,686,888–2,712,210	2,686,552	A	T	Promoter	2,686,750	2,686,750	-	A	Promoter
		2,686,719	G	A	Promoter	2,688,973	2,688,973	A	-	Intron
		2,695,032	C	T	Exon	2,689,483	2,689,483	A	-	Intron
		2,695,967	T	A	Intron	2,692,344	2,692,346	ATT	-	Intron
		2,699,145	C	T	Intron	2,692,712	2,692,712	-	A	Intron
		2,700,528	T	C	Intron	2,695,956	2,695,956	T	-	Intron
		2,700,850	Y	Y	Intron	2,699,669	2,699,669	T	-	Intron
		2,700,856	M	M	Intron	2,701,282	2,701,287	AAATGA	-	Intron
		2,701,386	Y	C	Intron	2,701,390	2,701,390	-	A	Intron
						2,701,394	2,701,394	-	C	Intron
						2,701,405	2,701,405	T	-	Intron
						2,702,933	2,702,933	A	-	Intron
						2,707,651	2,707,652	TA	-	Intron
						2,708,746	2,708,747	CT	-	3′UTR
						2,708,939	2,708,939	-	T	3′UTR
I21QA9	2,957,258–2,965,528					2,965,414	2,965,414	-	A	5′UTR
KNUI9Y	2,557,925–2,571,980	2,564,237	A	G	Intron	2,558,237	2,558,237	-	T	Intron
		2,564,258	T	C	Intron	2,558,253	2,558,253	-	G	Intron
						2,558,266	2,558,266	-	G	Intron
						2,558,352	2,558,352	-	T	Intron
						2,558,411	2,558,411	-	T	Intron
						2,558,514	2,558,514	-	C	5′UTR
						2,558,623	2,558,623	-	T	Intron
						2,558,673	2,558,673	-	G	Intron
						2,558,820	2,558,820	-	A	5′UTR
						2,558,855	2,558,855	-	G	5′UTR
						2,559,653	2,559,653	-	G	Exon
						2,561,414	2,561,414	A	-	Intron
						2,564,895	2,564,896	TC	-	Intron
						2,567,546	2,567,546	-	TCCA	Exon
L4W2C9	2,859,873–2,862,443	2,860,941	A	C	Intron	2,861,297	2,861,297	A	-	Intron
						2,861,302	2,861,314	AGCGGTTAATTAT	-	Intron
						2,862,221	2,862,221	-	CAT	Exon
LY2VHI	2,712,439–2,720,139	2,714,005	A	G	Exon					
		2,714,692	G	A	Intron					
WR01F1	2,843,963–2,851,619					2,849,340	2,849,340	T	-	Intron
						2,849,865	2,849,865	-	A	Intron
						2,851,435	2,851,435	-	A	Intron
WRGL83	2,721,576–2,723,325					2,721,863	2,721,863	A	-	Intron

**Table A4:** The expression patterns of genes in the co-localized genomic region.

Gene Name	2D2P15	657RUG	6XY8VG	D6UD48	EPQ3BP	GWX7Z5	H3KMIQ	I21QA9	KNUI9Y	L4W2C9	LY2VHI	M0GYTU	WR01F1	WRGL83	X8XU5X
CN0_SLH_1	5.7	1.7	4.6	4.9	0.2	18.0	3.9	6.6	14.0	0.4	17.4	3.6	2.9	1.0	15.5
CN0_SLH_2	4.8	5.2	3.9	10.1	0.1	17.8	5.4	12.2	25.5	0.5	24.4	4.9	4.7	1.3	20.0
CN0_SLH_3	4.9	3.4	4.4	8.1	0.3	20.4	4.8	10.6	23.4	0.5	21.2	5.9	4.1	1.4	15.4
CN0_JNH3_1	5.7	2.1	2.0	1.2	0.1	25.0	2.1	2.3	3.9	0.3	21.4	2.8	0.0	0.4	15.9
CN0_JNH3_2	4.4	2.2	3.0	2.0	0.1	24.4	2.0	2.6	5.6	0.2	22.0	3.2	0.0	0.3	27.4
CN0_JNH3_3	4.5	2.0	2.8	1.3	0.2	26.8	1.8	3.0	4.2	0.1	18.7	2.1	1.8	0.3	9.9
C1_SLH_1	22.6	23.1	7.5	10.9	2.9	83.9	15.2	27.0	20.3	11.4	24.4	5.9	5.3	6.6	34.6
C1_SLH_2	18.5	29.0	5.8	11.5	4.3	75.0	17.6	34.1	19.4	13.2	29.2	8.0	5.0	6.5	42.5
C1_SLH_3	25.3	20.9	5.2	9.9	2.7	74.0	14.7	26.1	17.9	13.3	27.1	6.8	6.4	8.4	39.2
C1_JNH3_1	41.1	15.8	5.3	11.9	1.9	68.6	14.6	29.0	15.9	8.8	35.4	8.0	7.5	7.8	30.4
C1_JNH3_2	51.8	10.6	7.7	8.6	1.6	73.8	13.0	17.2	12.9	5.6	25.4	11.9	5.2	6.0	26.7
C1_JNH3_3	42.3	14.9	6.0	8.6	1.7	67.0	13.0	22.2	14.9	12.2	31.5	9.7	6.5	5.8	29.9
N1_SLH_1	18.8	8.3	2.8	11.6	1.0	28.5	11.9	11.7	14.1	9.1	22.5	5.2	3.1	3.2	20.7
N1_SLH_2	16.8	12.1	3.8	12.4	1.8	22.0	13.7	15.3	12.4	12.1	30.0	3.7	6.1	4.3	27.0
N1_SLH_3	18.4	10.1	3.8	13.5	1.3	27.3	11.4	13.9	12.5	9.8	25.9	4.1	4.6	2.8	23.3
N1_JNH3_1	13.1	11.5	2.7	12.7	0.6	17.6	16.3	18.7	10.2	6.1	39.0	3.7	5.7	3.8	29.5
N1_JNH3_2	26.1	10.0	4.0	13.5	1.0	33.0	14.6	17.5	12.5	4.8	27.6	5.4	4.6	2.3	24.0
N1_JNH3_3	22.7	9.9	2.3	12.5	0.9	30.6	15.2	19.3	12.7	4.6	29.5	5.2	6.4	4.0	24.1
C2_SLH_1	16.5	25.6	7.2	20.9	2.7	83.7	17.4	35.4	14.8	18.6	34.1	7.8	5.7	3.4	40.9
C2_SLH_2	18.9	24.7	7.4	17.4	2.4	84.0	17.3	33.4	11.9	14.9	34.5	7.6	5.5	2.5	38.8
C2_SLH_3	22.8	20.8	6.5	15.9	2.1	89.1	14.8	30.0	14.1	13.9	30.2	8.5	5.0	3.1	36.4
C2_JNH3_1	55.9	11.8	5.7	10.8	1.3	77.6	13.3	25.0	13.5	7.7	34.1	8.1	6.7	4.7	31.7
C2_JNH3_2	57.6	11.8	9.1	9.4	1.2	76.9	13.5	27.7	17.3	8.8	31.1	8.2	5.5	4.5	30.9
C2_JNH3_3	54.4	10.8	6.5	11.6	0.9	82.3	12.4	23.1	12.3	6.9	30.5	8.2	5.5	5.4	30.8
N2_SLH_1	17.2	5.3	2.8	7.0	0.5	28.3	7.4	6.7	12.1	4.2	13.0	3.0	1.9	1.8	14.9
N2_SLH_2	18.8	6.6	2.8	8.6	0.9	22.2	7.6	7.6	11.3	5.2	15.3	4.3	2.5	2.1	18.4
N2_SLH_3	15.7	6.3	3.2	10.6	0.9	23.1	8.8	9.0	11.5	4.8	17.0	3.0	3.4	2.2	19.4
N2_JNH3_1	20.5	9.0	3.0	7.9	0.4	24.1	9.4	15.5	9.9	4.2	20.9	3.2	3.1	2.3	16.1
N2_JNH3_2	20.9	4.8	2.1	6.7	0.5	25.1	8.4	8.1	11.7	1.7	16.1	4.3	1.7	2.5	15.2
N2_JNH3_3	16.0	6.5	1.8	8.4	0.5	25.1	8.3	10.7	9.9	4.5	16.1	4.3	3.7	2.2	15.5
C3_SLH_1	20.8	17.9	7.5	19.5	1.2	66.6	12.1	25.3	13.8	6.7	33.2	7.9	4.4	1.8	26.5
C3_SLH_2	19.0	21.7	5.6	22.6	1.0	66.9	13.9	28.1	13.2	8.2	34.5	9.5	5.5	2.7	34.1
C3_SLH_3	17.2	22.0	7.3	21.4	1.4	62.6	14.3	26.7	14.1	9.1	39.2	7.0	5.1	2.0	29.4
C3_JNH3_1	53.1	10.0	5.0	15.9	1.3	75.0	15.1	25.6	15.0	6.8	29.7	11.2	4.7	2.9	26.6
C3_JNH3_2	42.6	14.9	5.5	19.8	2.3	70.2	15.6	35.9	13.9	8.5	48.7	12.9	6.8	2.6	34.6
C3_JNH3_3	45.2	15.6	5.6	18.5	1.9	72.5	17.4	36.2	18.1	7.4	49.5	11.7	8.2	4.8	36.4
N3_SLH_1	13.8	5.4	2.3	8.2	1.2	26.3	9.1	9.5	12.5	4.7	12.3	10.1	3.2	2.7	13.7
N3_SLH_2	14.4	5.1	2.6	8.1	1.2	22.6	8.9	10.0	11.3	2.0	11.8	9.0	3.1	3.4	13.6
N3_SLH_3	15.7	4.1	3.7	6.9	1.2	25.1	7.8	7.6	10.1	1.6	10.7	8.2	2.6	1.6	12.5
N3_JNH3_1	15.7	4.7	2.4	7.9	1.0	23.9	8.0	10.0	10.5	1.9	13.7	5.8	3.9	2.7	12.8
N3_JNH3_2	19.5	5.0	2.2	6.6	0.4	26.9	7.7	9.9	10.2	1.9	14.2	7.0	3.0	2.0	13.7
N3_JNH3_3	15.0	3.4	2.9	6.7	0.7	20.2	4.6	8.3	9.3	2.4	11.2	9.2	2.0	3.9	13.2
CN0_SLH	5.1	3.4	4.3	7.7	0.2	18.7	4.7	9.8	21.0	0.5	21.0	4.8	3.9	1.2	17.0
CN0_JNH3	4.9	2.1	2.6	1.5	0.1	25.4	2.0	2.6	4.6	0.2	20.7	2.7	0.6	0.4	17.7
C1_SLH	22.1	24.3	6.1	10.8	3.3	77.6	15.8	29.1	19.2	12.6	26.9	6.9	5.6	7.2	38.7
C1_JNH3	45.1	13.8	6.3	9.7	1.7	69.8	13.6	22.8	14.5	8.9	30.7	9.9	6.4	6.5	29.0
N1_SLH	18.0	10.2	3.5	12.5	1.4	25.9	12.3	13.6	13.0	10.3	26.1	4.3	4.6	3.4	23.7
N1_JNH3	20.6	10.5	3.0	12.9	0.9	27.1	15.4	18.5	11.8	5.2	32.0	4.7	5.6	3.3	25.9
C2_SLH	19.4	23.7	7.0	18.0	2.4	85.6	16.5	32.9	13.6	15.8	33.0	8.0	5.4	3.0	38.7
C2_JNH3	55.9	11.4	7.1	10.6	1.1	78.9	13.1	25.3	14.4	7.8	31.9	8.2	5.9	4.9	31.1
N2_SLH	17.2	6.1	3.0	8.7	0.8	24.5	7.9	7.8	11.6	4.7	15.1	3.4	2.6	2.1	17.6
N2_JNH3	19.1	6.7	2.3	7.6	0.5	24.8	8.7	11.4	10.5	3.5	17.7	3.9	2.8	2.3	15.6
C3_SLH	19.0	20.5	6.8	21.2	1.2	65.4	13.4	26.7	13.7	8.0	35.6	8.1	5.0	2.2	30.0
C3_JNH3	46.9	13.5	5.4	18.1	1.8	72.6	16.0	32.6	15.7	7.6	42.6	11.9	6.6	3.4	32.5
N3_SLH	14.6	4.9	2.9	7.7	1.2	24.7	8.6	9.0	11.3	2.8	11.6	9.1	3.0	2.6	13.3
N3_JNH3	16.7	4.4	2.5	7.1	0.7	23.7	6.7	9.4	10.0	2.1	13.0	7.4	3.0	2.9	13.2

**Table A5:** The details of the alleles of the 60 lines from the RIL population by developed KASP markers.

Number	Line Number	Genotype	Phenotype
1	293	AA	cold-tolerance
2	295	AA	cold-tolerance
3	300	AA	cold-tolerance
4	301	AA	cold-tolerance
5	303	AA	cold-tolerance
6	311	CC	cold-tolerance
7	318	AA	cold-tolerance
8	325	AA	cold-tolerance
9	326	AA	cold-tolerance
10	332	CC	cold-tolerance
11	336	AA	cold-tolerance
12	370	AA	cold-tolerance
13	385	AA	cold-tolerance
14	393	AA	cold-tolerance
15	405	CC	cold-tolerance
16	430	AA	cold-tolerance
17	448	AA	cold-tolerance
18	479	AA	cold-tolerance
19	484	AA	cold-tolerance
20	487	CC	cold-tolerance
21	489	AA	cold-tolerance
22	509	AA	cold-tolerance
23	538	AA	cold-tolerance
24	292	CC	cold-sensitive
25	344	-	cold-sensitive
26	355	CC	cold-sensitive
27	360	CC	cold-sensitive
28	368	CC	cold-sensitive
29	372	CC	cold-sensitive
30	381	AA	cold-sensitive
31	384	CC	cold-sensitive
32	401	CC	cold-sensitive
33	403	CC	cold-sensitive
34	407	AA	cold-sensitive
35	416	CC	cold-sensitive
36	422	CC	cold-sensitive
37	432	AA	cold-sensitive
38	439	CC	cold-sensitive
39	452	CC	cold-sensitive
40	454	CC	cold-sensitive
41	455	CC	cold-sensitive
42	456	CC	cold-sensitive
43	461	CC	cold-sensitive
44	467	AA	cold-sensitive
45	469	CC	cold-sensitive
46	498	CC	cold-sensitive
47	507	CC	cold-sensitive
48	508	CC	cold-sensitive
49	510	CC	cold-sensitive
50	514	CC	cold-sensitive
51	517	AA	cold-sensitive
52	518	CC	cold-sensitive
53	521	CC	cold-sensitive
54	522	CC	cold-sensitive
55	523	AA	cold-sensitive
56	524	CC	cold-sensitive
57	525	CC	cold-sensitive
58	526	CC	cold-sensitive
59	534	CC	cold-sensitive
60	535	CC	cold-sensitive

**Table A6:** Environmental conditions during the germination period.

City	Date	Temperature_Mean_c	Temperature_Min_c	Temperature_Max_c	Precipitation_mm	Relative_Humidity_Mean_Pct	Relative_Humidity_Min_Pct	Relative_Humidity_Max_Pct
Baoding	15 April 2021	17.0	11.4	26.0	0.0	30.3	14.0	50
Baoding	16 April 2021	14.4	8.5	19.9	0.0	19.7	11.0	28
Baoding	17 April 2021	14.5	8.5	21.4	0.0	20.3	11.0	33
Baoding	18 April 2021	17.0	7.9	25.6	0.0	26.7	14.0	42
Baoding	19 April 2021	20.3	11.1	28.8	0.0	34.5	20.0	49
Baoding	20 April 2021	21.3	14.8	27.4	0.0	40.1	22.0	63
Baoding	21 April 2021	17.6	13.7	20.5	0.0	54.4	39.0	74
Baoding	22 April 2021	15.9	12.3	19.0	3.3	66.8	53.0	91
Baoding	23 April 2021	16.2	10.7	21.9	0.0	65.7	48.0	86
Baoding	24 April 2021	16.9	11.8	20.6	0.0	62.3	50.0	82
Baoding	25 April 2021	16.7	14.3	19.8	0.0	61.4	49.0	79
Baoding	26 April 2021	17.6	13.8	22.5	0.2	52.8	41.0	74
Baoding	27 April 2021	19.0	11.8	25.5	0.0	30.4	11.0	82
Baoding	28 April 2021	18.1	10.4	23.0	0.0	21.6	12.0	40
Baoding	29 April 2021	18.0	11.5	23.3	0.0	22.9	13.0	39
Baoding	30 April 2021	12.9	10.3	16.3	0.2	40.7	26.0	62
Baoding	1 May 2021	17.0	10.0	23.7	0.0	32.2	12.0	62
Baoding	2 May 2021	15.8	6.1	21.9	0.0	30.7	18.0	51
Baoding	3 May 2021	18.3	13.9	22.3	0.0	45.6	35.0	63
Baoding	4 May 2021	19.7	13.7	25.0	0.0	29.0	11.0	72
Baoding	5 May 2021	21.3	11.2	29.4	0.0	22.6	11.0	43
Baoding	6 May 2021	23.6	18.7	30.7	0.0	18.5	9.0	30
Baoding	7 May 2021	21.4	15.3	26.7	0.0	23.7	16.0	34
Baoding	8 May 2021	23.4	17.1	27.8	0.0	19.8	13.0	26
Baoding	9 May 2021	22.2	15.7	28.5	0.0	22.9	13.0	35
Baoding	10 May 2021	21.1	15.6	25.4	0.0	32.7	24.0	56
Baoding	11 May 2021	19.2	12.9	24.9	0.0	52.5	35.0	73
Baoding	12 May 2021	21.7	15.0	27.7	0.0	52.8	37.0	74
Baoding	13 May 2021	23.0	18.0	28.1	0.0	61.3	42.0	79
Baoding	14 May 2021	22.3	19.2	25.9	0.0	70.8	59.0	81
Baoding	15 May 2021	18.6	16.0	21.4	0.9	69.5	41.0	85
Baoding	16 May 2021	19.4	14.7	24.6	0.9	46.8	27.0	67
Baoding	17 May 2021	23.1	13.6	31.4	0.0	38.6	17.0	70
Baoding	18 May 2021	24.9	16.1	32.8	0.0	36.7	19.0	58
Baoding	19 May 2021	24.6	17.6	31.8	0.0	49.8	28.0	73
Baoding	20 May 2021	22.6	18.8	27.5	1.0	65.5	44.0	91
Baoding	15 April 2022	14.7	10.2	19.2	0.0	24.8	13.0	40
Baoding	16 April 2022	15.5	6.4	22.7	0.0	26.8	13.0	45
Baoding	17 April 2022	16.2	10.3	21.7	0.0	34.8	25.0	42
Baoding	18 April 2022	17.0	12.8	22.6	0.0	45.5	31.0	61
Baoding	19 April 2022	19.4	10.6	27.3	0.0	39.9	17.0	70
Baoding	20 April 2022	21.6	11.8	28.9	0.0	35.0	18.0	60
Baoding	21 April 2022	23.6	18.6	29.4	0.0	31.4	21.0	44
Baoding	22 April 2022	17.8	13.3	21.8	0.0	31.0	25.0	44
Baoding	23 April 2022	20.6	11.5	29.5	0.0	39.2	23.0	56
Baoding	24 April 2022	22.2	16.7	27.8	0.0	51.7	38.0	67
Baoding	25 April 2022	22.3	16.3	29.5	0.0	69.8	50.0	88
Baoding	26 April 2022	21.9	17.3	25.5	0.0	31.3	11.0	89
Baoding	27 April 2022	15.3	11.6	17.5	0.0	30.9	15.0	44
Baoding	28 April 2022	11.1	8.4	14.4	6.1	74.0	46.0	92
Baoding	29 April 2022	13.5	7.0	18.9	0.0	72.5	52.0	94
Baoding	30 April 2022	17.4	13.2	22.0	0.0	29.3	15.0	86
Baoding	1 May 2022	19.2	9.2	28.1	0.1	32.8	16.0	63
Baoding	2 May 2022	20.4	10.7	28.0	0.0	44.3	22.0	80
Baoding	3 May 2022	24.6	15.3	33.3	0.0	34.4	17.0	55
Baoding	4 May 2022	26.1	16.9	33.7	0.0	32.7	18.0	53
Baoding	5 May 2022	26.2	21.4	31.6	0.0	39.3	31.0	53
Baoding	6 May 2022	17.2	11.6	23.4	2.8	56.8	33.0	76
Baoding	7 May 2022	11.0	8.3	15.5	14.3	77.4	38.0	94
Baoding	8 May 2022	10.2	8.0	12.5	8.8	84.3	69.0	97
Baoding	9 May 2022	12.7	9.3	16.6	0.0	72.8	54.0	91
Baoding	10 May 2022	13.3	8.7	18.3	0.4	78.6	56.0	95
Baoding	11 May 2022	15.9	13.2	20.0	3.6	78.0	51.0	92
Baoding	12 May 2022	14.1	11.5	17.3	11.9	78.9	60.0	93
Baoding	13 May 2022	16.3	8.5	22.8	0.0	51.5	21.0	94
Baoding	14 May 2022	17.1	8.7	24.0	0.0	47.4	22.0	73
Baoding	15 May 2022	19.4	10.0	26.9	0.0	44.5	20.0	77
Baoding	16 May 2022	21.6	11.0	29.3	0.0	35.7	16.0	67
Baoding	17 May 2022	22.8	14.0	31.4	0.0	43.1	29.0	69
Baoding	18 May 2022	24.0	18.4	29.9	0.0	41.4	22.0	70
Baoding	19 May 2022	23.3	17.8	28.6	0.0	54.4	42.0	64
Baoding	20 May 2022	24.0	16.0	31.0	0.0	56.8	37.0	79
Fuxin	25 April 2021	11.9	4.5	18.5	0.0	32.7	18.0	49
Fuxin	26 April 2021	15.2	10.5	21.3	0.0	30.7	17.0	58
Fuxin	27 April 2021	14.5	10.6	19.5	0.0	46.0	19.0	79
Fuxin	28 April 2021	14.0	8.4	18.4	0.0	25.3	15.0	42
Fuxin	29 April 2021	11.7	4.5	16.3	0.3	44.8	35.0	68
Fuxin	30 April 2021	9.4	0.4	16.5	0.0	46.8	21.0	79
Fuxin	1 May 2021	9.7	3.3	14.9	0.1	47.3	24.0	86
Fuxin	2 May 2021	14.1	5.9	20.9	0.0	36.8	18.0	63
Fuxin	3 May 2021	15.1	10.6	21.7	0.0	48.1	34.0	68
Fuxin	4 May 2021	14.5	11.0	20.1	1.4	50.1	19.0	84
Fuxin	5 May 2021	16.5	8.9	23.0	0.0	23.4	11.0	46
Fuxin	6 May 2021	18.0	9.6	21.8	4.0	42.1	22.0	83
Fuxin	7 May 2021	13.8	7.9	20.6	0.7	47.9	26.0	76
Fuxin	8 May 2021	13.6	10.4	18.7	0.4	42.8	31.0	60
Fuxin	9 May 2021	15.9	8.3	22.7	0.0	34.4	16.0	54
Fuxin	10 May 2021	16.7	9.5	23.4	0.1	44.5	27.0	69
Fuxin	11 May 2021	18.0	10.1	24.7	0.0	42.4	21.0	71
Fuxin	12 May 2021	19.7	15.0	25.2	0.0	33.9	13.0	53
Fuxin	13 May 2021	17.3	15.0	20.4	0.8	67.5	50.0	94
Fuxin	14 May 2021	19.9	14.6	25.7	0.4	70.6	49.0	96
Fuxin	15 May 2021	15.2	12.5	17.9	8.6	52.9	25.0	87
Fuxin	16 May 2021	17.6	11.6	24.1	0.0	37.8	19.0	58
Fuxin	17 May 2021	20.5	7.9	29.6	0.0	31.9	11.0	64
Fuxin	18 May 2021	22.2	11.3	30.6	0.9	40.8	21.0	76
Fuxin	19 May 2021	23.3	15.0	30.9	0.0	44.5	27.0	66
Fuxin	20 May 2021	20.9	14.0	26.5	0.0	61.8	42.0	89
Fuxin	21 May 2021	20.7	12.5	28.9	0.0	64.8	39.0	91
Fuxin	22 May 2021	22.8	15.4	30.0	0.0	63.6	41.0	90
Fuxin	23 May 2021	17.9	12.4	22.3	0.8	50.6	19.0	87
Fuxin	24 May 2021	17.0	11.8	21.4	0.0	24.5	12.0	49
Fuxin	25 May 2021	18.3	11.3	24.4	0.0	24.1	10.0	45
Fuxin	26 May 2021	14.2	9.4	18.0	2.3	64.3	34.0	88
Fuxin	27 May 2021	15.7	9.0	21.9	3.6	65.5	33.0	92
Fuxin	28 May 2021	17.1	12.6	22.5	3.4	62.5	30.0	95
Fuxin	29 May 2021	18.4	10.0	25.4	0.0	49.1	22.0	81
Fuxin	30 May 2021	18.3	14.3	22.1	0.0	54.2	36.0	74
Fuxin	15 April 2022	10.8	4.9	16.9	0.0	29.0	12.0	59
Fuxin	16 April 2022	14.0	6.7	20.5	0.0	20.1	12.0	33
Fuxin	17 April 2022	14.8	8.2	22.2	0.0	26.1	12.0	40
Fuxin	18 April 2022	13.8	6.4	19.3	0.0	29.0	13.0	54
Fuxin	19 April 2022	18.0	8.9	26.4	0.0	33.3	19.0	48
Fuxin	20 April 2022	18.4	13.2	24.5	0.0	40.1	30.0	55
Fuxin	21 April 2022	14.9	7.5	19.5	6.6	64.9	50.0	91
Fuxin	22 April 2022	12.7	6.6	18.5	0.0	43.6	16.0	76
Fuxin	23 April 2022	14.8	6.5	21.0	0.0	49.8	27.0	75
Fuxin	24 April 2022	16.3	10.6	21.9	0.0	39.7	24.0	67
Fuxin	25 April 2022	16.1	8.9	22.9	0.0	74.3	42.0	90
Fuxin	26 April 2022	14.8	10.0	17.8	0.0	41.8	12.0	91
Fuxin	27 April 2022	12.2	6.5	15.7	0.0	23.4	9.0	41
Fuxin	28 April 2022	12.7	7.3	17.8	0.0	33.3	21.0	56
Fuxin	29 April 2022	12.2	3.3	18.7	0.6	45.3	19.0	75
Fuxin	30 April 2022	12.2	7.9	16.7	0.0	24.5	11.0	51
Fuxin	1 May 2022	11.4	4.7	17.1	0.0	26.7	15.0	45
Fuxin	2 May 2022	12.2	1.3	20.2	0.5	29.2	14.0	57
Fuxin	3 May 2022	19.2	10.8	29.2	0.9	42.2	22.0	66
Fuxin	4 May 2022	21.2	13.1	29.1	0.0	49.2	29.0	80
Fuxin	5 May 2022	21.7	16.4	25.7	1.4	43.5	34.0	67
Fuxin	6 May 2022	15.3	12.0	18.7	0.4	35.5	13.0	74
Fuxin	7 May 2022	14.1	8.1	18.9	0.0	20.8	10.0	39
Fuxin	8 May 2022	15.0	9.5	20.8	0.0	31.4	16.0	60
Fuxin	9 May 2022	16.3	7.3	23.8	0.0	37.4	14.0	72
Fuxin	10 May 2022	14.6	10.0	19.5	0.1	55.2	38.0	70
Fuxin	11 May 2022	13.3	6.8	19.8	0.1	50.4	17.0	84
Fuxin	12 May 2022	14.1	7.6	19.2	0.0	30.3	17.0	71
Fuxin	13 May 2022	13.4	5.6	19.5	0.3	32.3	17.0	58
Fuxin	14 May 2022	13.6	8.2	19.3	0.0	28.2	14.0	42
Fuxin	15 May 2022	16.9	8.1	23.2	0.2	31.5	18.0	53
Fuxin	16 May 2022	18.3	12.6	24.1	0.0	28.8	12.0	46
Fuxin	17 May 2022	20.0	13.1	27.3	0.4	34.6	18.0	55
Fuxin	18 May 2022	21.6	14.5	28.2	0.3	35.9	13.0	64
Fuxin	19 May 2022	22.4	15.6	29.5	1.0	39.3	20.0	77
Fuxin	20 May 2022	22.0	11.2	30.6	0.0	52.7	17.0	95
Fuxin	21 May 2022	23.8	16.0	30.5	0.8	51.7	27.0	90
Fuxin	22 May 2022	22.8	16.1	29.0	0.0	47.5	22.0	94
Fuxin	23 May 2022	24.8	16.9	32.4	1.9	47.5	31.0	69
Fuxin	24 May 2022	23.1	11.2	30.3	5.9	52.5	32.0	88
